# Elucidation of the Pathway for the Biosynthesis of the Undecorated Capsular Polysaccharide from the HS:19 Serotype of the Human Pathogen *Campylobacter jejuni*

**DOI:** 10.1021/acs.biochem.6c00264

**Published:** 2026-06-04

**Authors:** Dao Feng Xiang, Tamari Narindoshvili, Frank M. Raushel

**Affiliations:** Department of Chemistry, 14736 Texas A&M University, College Station, Texas 77842, United States

## Abstract

*Campylobacter jejuni* is the leading cause of food poisoning in the United States and Europe. The bacterium is commensal in poultry, and the ingestion of contaminated or undercooked chicken products can result in rather severe cases of nausea, abdominal pain, and diarrhea. Protecting the bacterium from the host immune system is a thick polysaccharide coat known as the capsular polysaccharide. The repeating disaccharide unit in the undecorated capsular polysaccharide from the HS:19 serotype of *C. jejuni* consists of N-acetyl-d-glucosamine (GlcNAc) and the serinol amide of d-glucuronic acid (GlcA). It was demonstrated that the GT2 glycosyltransferase from the N-terminal domain of HS19.08 catalyzes the transfer of d-glucuronic acid from UDP-GlcA to C3 of the GlcNAc moiety at the nonreducing end of the growing polysaccharide chain. The carboxylate of the added GlcA is then amidated by the ATP-dependent ligase activity of the C-terminal domain of HS19.11 using (*S*)-serinol-phosphate. The phosphorylated serinol amide is subsequently dephosphorylated by the catalytic activity of HS19.09. In the final step of the series, the GT2 glycosyltransferase from the N-terminal domain of HS19.11 catalyzes the transfer of GlcNAc from UDP-GlcNAc to C4 of the amidated glucuronic acid. This series of reactions has been exploited to enzymatically synthesize and isolate defined oligomers containing 2, 3, 5, and 7 monomeric units, and procedures were developed for the synthesis of oligomeric mixtures ranging from 9 to 15 monomeric units.

## Graphical abstract

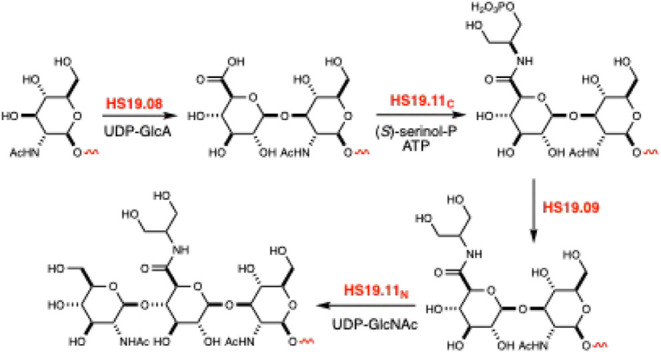

## Introduction

*Campylobacter jejuni* is the leading cause of food poisoning in the United States and Europe.^1–4^ The bacterium is commensal in poultry and the ingestion of contaminated or undercooked chicken products can result in rather severe cases of nausea, abdominal pain and diarrhea.^5^ It has also been noted that a significant fraction of patients with Guillain-Barré Syndrome have had a prior *C. jejuni* infection.^6–9^ Protecting the bacterium from the host-immune system is a thick polysaccharide coat known as the capsular polysaccharide (CPS).^10^ The CPS consists of a repeating sequence of 2–5 different carbohydrates that can exceed 100 units in length. It is currently thought that the polysaccharide is attached to a poly-KDO (3-deoxy-d-*manno*-oct-2-ulosonic acid) linker that is, in turn, attached to a diacylglycerophosphate anchor that is embedded in the outer bacterial membrane.^11,12^ To date, at least 33 different serotypes of *C. jejuni* have been identified and the gene loci for the enzymes needed for CPS formation have been identified.^13–15^ The structures of the repeating carbohydrate sequence for at least 12 of these serotypes have been chemically characterized.^16,17^

The undecorated capsular polysaccharide from the HS:02 serotype of *C. jejuni* consists of the serinol amide of d-glucuronic acid (GlcA), N-acetyl-d-galactosamine (GalNAc) and d-ribose (Rib).^18^ The polysaccharide is further decorated with d-*glycero*-l-*gluco*-heptose, methylphosphoramidate, and methylation as illustrated in Figure 1a.^19,20^ The reported gene cluster that is required to produce the enzymes needed for the biosynthesis of the CPS from the HS:02 serotype is presented in Figure 2a where 28 genes reside between those for KspC and KspF.^21,22^ We have recently identified, and subsequently characterized, the three glycosyltransferases needed for the biosynthesis of the undecorated CPS from the HS:02 serotype of *C. jejuni*. The N-terminal domain of Cj1432 (Cj1432_N_) is required to transfer d-glucuronic acid from UDP-GlcA to the C2-hydroxyl of the d-ribose moiety.^23^ The N-terminal domain of either Cj1438 (Cj1438_N_) or Cj1434 (Cj1434_N_) is utilized for the transfer of N-acetyl-d-galactosamine from UDP-GalNAc to the C4-hydroxyl group of the GlcA moiety.^24^ Finally, the C-terminal domain of Cj1432 (Cj1432_C_) is needed to transfer d-ribose-5-phosphate from phosphoribosyl pyrophosphate (PRPP) to the C5-hydroxyl of the GalNAc moiety.^25^ In addition to these three glycosyltransferase-catalyzed reactions, the C-terminal domain of Cj1438 (Cj1438_C_) is necessary for the ATP-dependent amidation of the GlcA moiety with (*S*)-serinol-phosphate, and Cj1435 is employed to hydrolyze the phosphate from this product.^19^ An additional phosphatase from the middle domain of Cj1432 (Cj1432_M_) is utilized to hydrolyze phosphate from the ribose-5-phosphate intermediate after the reaction catalyzed by Cj1432_C_. These reactions are summarized in Figure 3.

**Figure 1:**
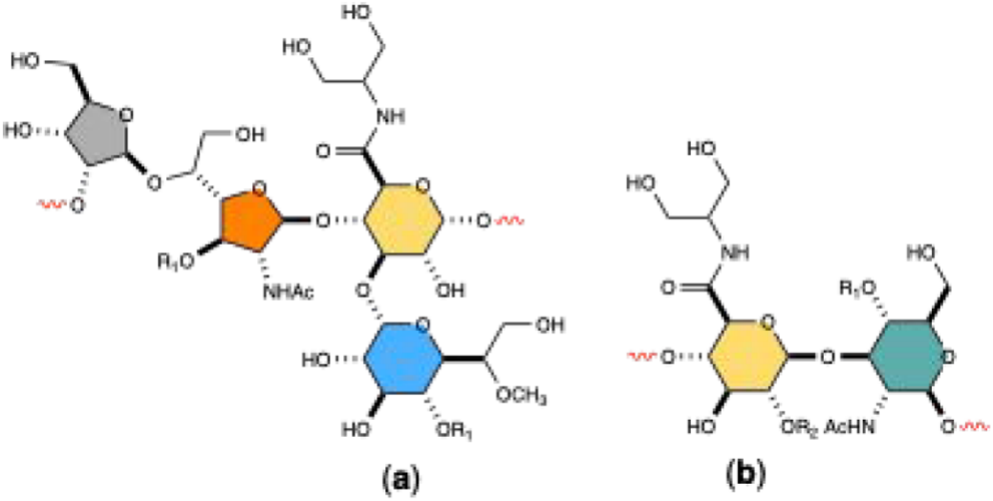
Structures of the repeating capsular polysaccharides from the HS:02 and HS:19 serotypes of *C. jejuni*. (a) HS:02 serotype consisting of d-glucuronic acid (depicted in yellow); N-acetyl-d-galactosamine (depicted in orange); d-ribose (depicted in gray) and d-*glycero*-l-*gluco*-heptose (depicted in blue). (b) HS:19 serotype consisting of d-glucuronic acid (depicted in yellow) and N-acetyl-d-glucosamine (depicted in green). The R_1_ adduct is methyl phosphoramidate (MeOPN) and the R_2_ adduct is l-sorbose.

**Figure 2:**
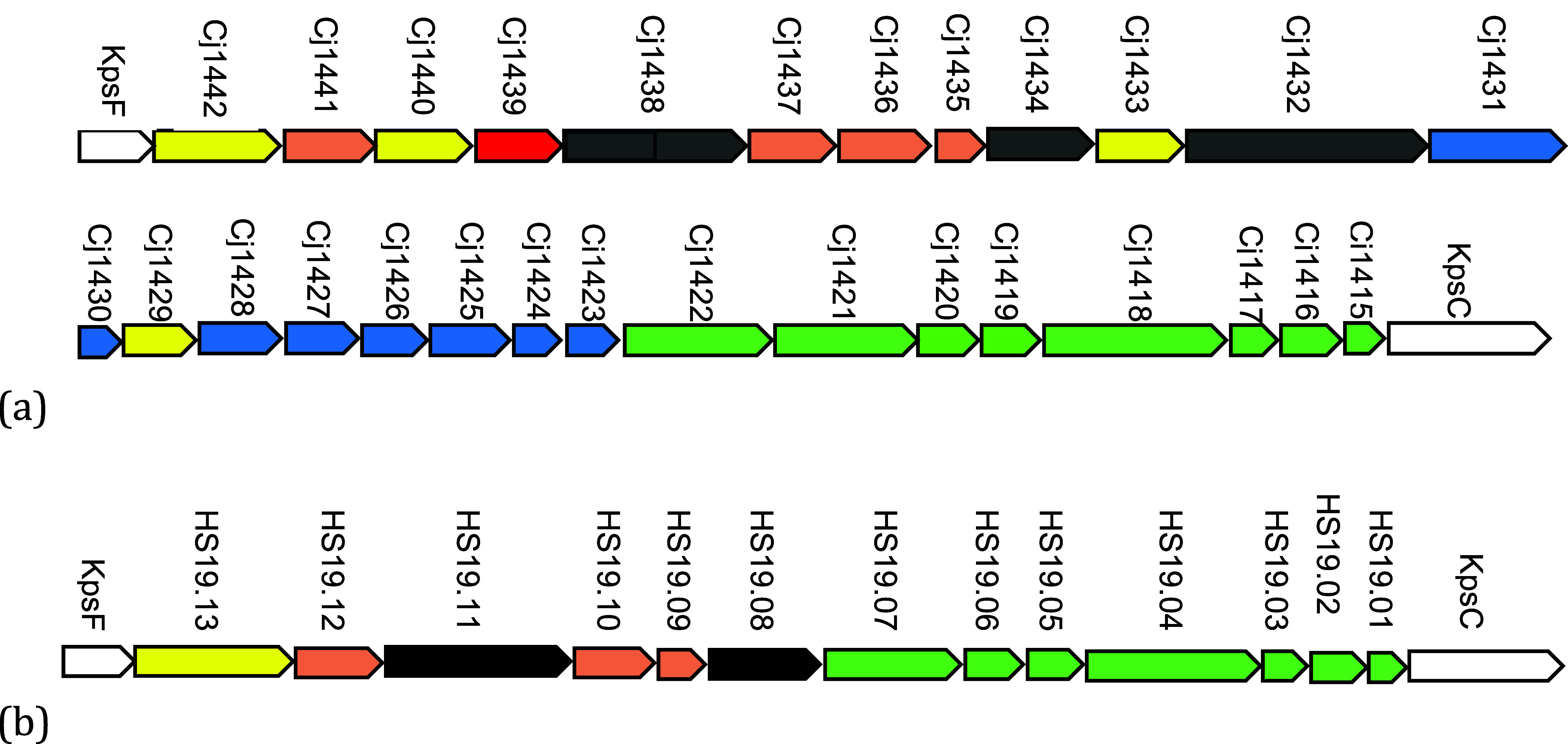
Associated gene clusters for the biosynthesis of the CPS from the HS:02 and HS:19 serotypes of *C. jejuni*. (a) Gene cluster for the HS:02 serotype; (b) Gene cluster for the HS:19 serotype. In both clusters the genes colored green are associated with the biosynthesis and subsequent transfer of the methyl phosphoramidate (MeOPN) decoration; those colored blue are associated with the biosynthesis and transfer of d-*glycero*-l-*gluco*-heptose; those colored salmon are associated with the biosynthesis of UDP-GlcA and the formation of (*S*)-serinol-phosphate and ethanolamine phosphate. The black colored genes encode the polymerizing glycosyltransferases. Cj1439 (colored red) is a pyranose/furanose mutase. The genes colored yellow are of an unknown function.

**Figure 3:**
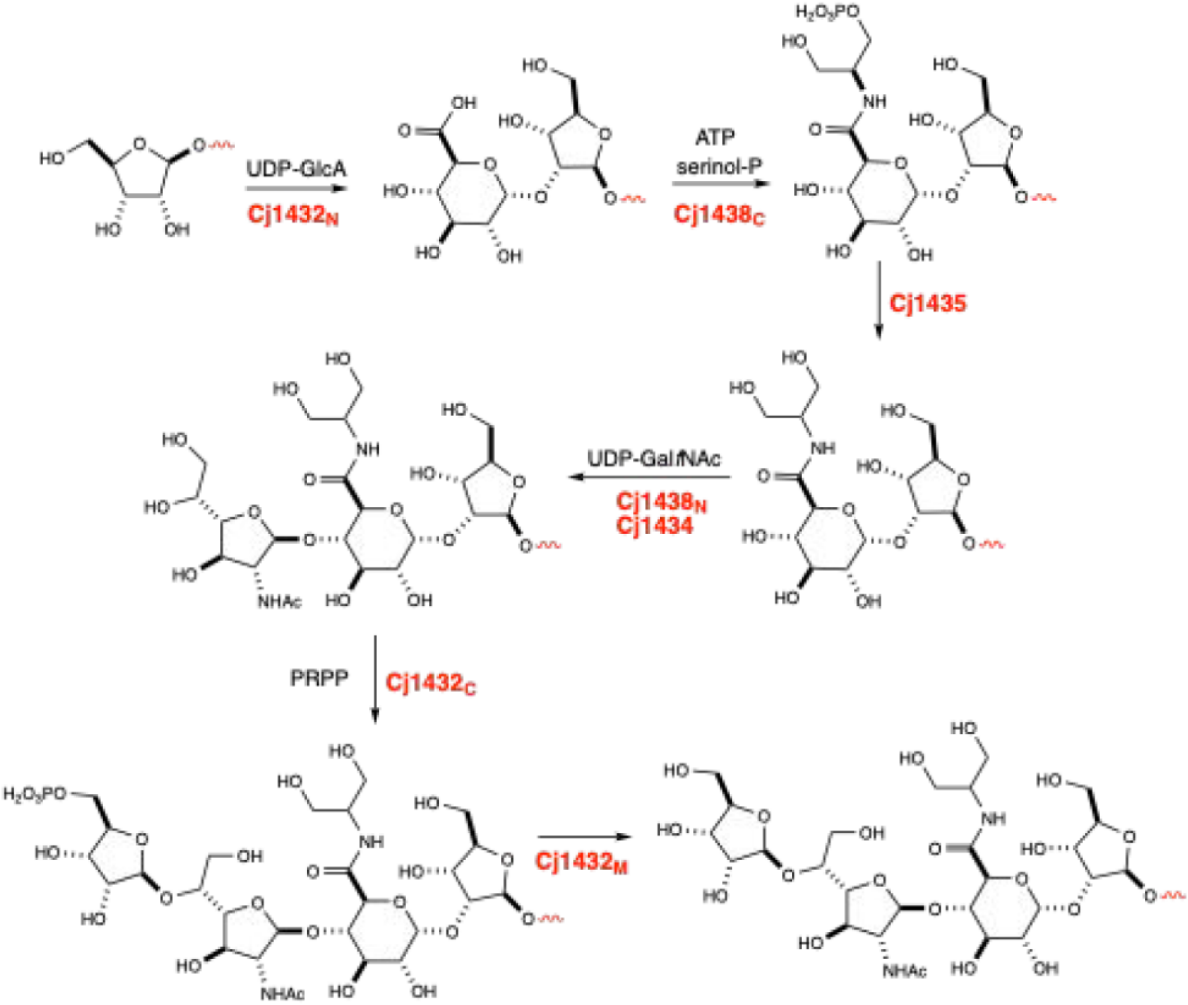
Sequence of six enzyme-catalyzed reactions required for the polymerization of the CPS from the HS:02 serotype of *C. jejuni*. The reaction pathway starts arbitrarily with the addition of d-glucuronic acid to d-ribose at the nonreducing end of the growing CPS.

The repeating carbohydrate sequence of the CPS from the HS:19 serotype is presented in Figure 1b.^26,27^ It is somewhat simpler than the one determined for the HS:02 serotype having a repeating disaccharide sequence of d-glucuronic acid and N-acetyl-d-glucosamine. The GlcA moiety is amidated with serinol and decorated with l-sorbose, while the GlcNAc moiety is modified with methyl phosphoramidate. The gene cluster for the expression of the genes required for the biosynthesis of this CPS is shown in Figure 2b. Here one can easily identify the 7 genes associated with the biosynthesis and transfer of the MeOPN decoration (HS19.01 through HS19.07). Since this CPS also contains a GlcA moiety amidated with serinol it is likely that enzymes similar to those found in the HS:02 serotype will be required for the generation of UDP-GlcA, serinol-P, and the associated phosphatase. Preliminary examination of the corresponding gene locus suggests that these reactions will be catalyzed by HS19.12, HS19.10, and HS19.09, respectively. Of more immediate interest is the identification of the two glycosyltransferases needed for polymerization of GlcA and GlcNAc. Unlike the polymerases identified for the HS:02 serotype, both glycosyltransferases are expected to be GT2 transferases since the product stereochemistry must occur with an inversion of configuration.^26,27^ Here we have demonstrated that the N-terminal domain of HS19.08 catalyzes the transfer of GlcA from UDP-GlcA to the C3-hydroxyl of GlcNAc and that the N-terminal domain of HS19.11 catalyzes the transfer of GlcNAc to the C4-hydroxyl group of the serinol amide of GlcA.

## Materials Methods

### Materials

Adenosine 5′-triphosphate (ATP), UDP-d-glucuronic acid (UDP-GlcA), UDP-N-acetylglucosamine (UDP-GlcNAc), and methyl-β-d-glucuronic acid (**1a**), were purchased from Sigma-Aldrich. Methyl 2-acetamido-2-deoxy-β-d-glucopyranoside (**5**) was purchased from Combi-Blocks. Growth medium lysogeny broth (LB) and isopropyl-β-d-thiogalactopyranoside (IPTG) were purchased from Research Products International. HisTrap columns, HiTrap Q HP anion exchange columns, and Vivaspin 20 10 kDa molecular weight cutoff (MWCO) spin filters were obtained from Cytiva. The 10K Nanosep spin filters were purchased from PALL Corporation (Port Washington, NY). The protease inhibitor cocktail (cOmplete Mini), DNase I, kanamycin, gentamicin, ampicillin, imidazole, and HEPES were purchased from Sigma-Aldrich. All other compounds, unless stated otherwise, were purchased from either Sigma-Aldrich or Thermo Fisher Scientific. Methyl-[^13^C-4]-α/β-d-glucuronic acid (**1b**) and (*S*)-serinol phosphate were chemically synthesized as previously reported.^24,25^

### Equipment

The purification of proteins and reaction products were carried out using an NGC Chromatography System (Bio-Rad). Ultraviolet spectra were collected using a SpectraMax ABS Plus UV–vis plate reader (Molecular Devices) with a 1 cm quartz cuvette. Nuclear magnetic resonance (NMR) spectra were obtained at 30 °C using standard pulse sequences on an Avance III 500 MHz NMR spectrometer equipped with a broad band probe and sample changer. Electrospray ionization mass spectrometry (ESI-MS) experiments were performed using a Thermo Scientific Q Exactive Focus instrument. Samples were injected into a 10 μL loop and transferred to the instrument using a mobile phase containing 70% methanol and 30% water with 0.1% formic acid at a flow rate of 600 μL/min. The Q Exactive Focus HESI source was operated in full MS in positive and negative modes. The mass resolution was tuned to 70,000 fwhm (full width at half-maximum) at *m*/*z* 200. The spray voltage was set to 3.5 kV for positive mode and 2.8 kV for negative mode. The vaporizer and transfer capillary temperatures were held at 250 and 320 °C, respectively. The S-Lens RF level was set at 50 V. Exactive Series 2.11/Xcalibur 4.2.47 software was used for data acquisition and processing. The predicted *m*/*z* values for the various compounds isolated in this investigated were determined using the software available within ChemDraw.

### Plasmid Construction

The genes for HS19.08 and HS19.11 employed in this study are from *C. jejuni* NCTC 12517 (serotype HS:19). The gene for Cj1435 (UniProt ID: Q0P8H9) is from *C. jejuni* NCTC 11168 (serotype HS:02). The N-terminal truncated gene for HS19.08 (residues 1–381; UniProt ID: Q5M6M5, and denoted here as HS19.08_N_), the N-terminal truncated gene for HS19.11 (residues 1–384; UniProt ID: Q5M6M2, and denoted here as HS19.11_N_), and the C-terminal truncated gene for HS19.11 (residues 513–832; UniProt ID: Q5M6M2, and denoted here as HS19.11_C_) were chemically synthesized and cloned into a pET28a expression vector containing an N-terminal hexahistidine tag by TWIST Bioscience, USA, respectively. The isolation and purification of Cj1435 has been previously described.^19,24^ The amino acid sequences for HS19.08_N_, HS19.11_N_, and HS19.11_C_ are shown in Figure S1.^28^

### Purification of HS19.08_N_

The plasmid DNA containing the gene for HS19.08_N_ was used to transform *Eschericha coli* BL21­(DE3) competent cells, and single colonies were used to create starter cultures that contained kanamycin (50 μg/mL). The starter cultures were used to inoculate 1.0 L of LB medium. The cultures were allowed to grow at 37 °C until an OD_600_ of 0.6–0.8 was reached, and protein expression was induced by the addition of 1.0 mM IPTG. The cultures were grown at 20 °C for 18 h and then harvested by centrifugation (7000 rcf, 4 °C, 10 min). The resulting cell pellet was flash frozen in liquid nitrogen and stored at −80 °C. For purification of HS19.08_N_, 10 g of frozen cells were resuspended in 100 mL of 50 mM HEPES, 250 mM NaCl, 10 mM imidazole, pH 8.0, containing 0.05 mg/mL of a protease inhibitor cocktail and 40 U/mL of DNase I. The resuspended cells were lysed by sonication (QSONICA Sonicator Ultrasonic Processor) in an ice bath. The lysate was clarified by centrifugation at 18,000*g* and 4 °C for 30 min. The clarified supernatant was passed through a 0.45 μm syringe filter (Whatman) and loaded onto a 5 mL HisTrap column (GE Healthcare) connected to an NGC liquid chromatography system (Bio-Rad) previously equilibrated with binding buffer (50 mM HEPES, 250 mM NaCl, 10 mM imidazole, pH 8.0). The His-tagged protein was isolated using a 0–50% gradient of elution buffer (50 mM HEPES, 0.25 M NaCl, 0.50 M imidazole, pH 8.0). Fractions containing the desired protein, as identified by SDS-PAGE, were combined, and the imidazole was removed by dialysis against a buffer containing 50 mM HEPES and 250 mM NaCl (pH 8.0). The protein was concentrated to ∼10 mg/mL, aliquoted, flash-frozen in liquid nitrogen, and stored at −80 °C. The protein concentration was determined spectrophotometrically using a computationally derived molar extinction coefficient at 280 nm.^29^ The molecular weight and molar extinction coefficient (ε280) of HS19.08_N_ were 47 947 Da and 77,615 M^–1^ cm^–1^, respectively. Approximately 28 mg of enzyme were obtained per liter of cell culture.

### Purification of HS19.11_N_, HS19.11_C_, and Cj1435

The enzymes HS19.11_N_ and HS19.11_C_ were expressed and purified under the same cell growth conditions and using the same protein purification procedures as described for HS19.08_N_. The molecular weight and molar extinction coefficient (ε280) of HS19.11_N_ were calculated to be 48 263 Da and 67,645 M^–1^ cm^–1^, respectively.^29^ Approximately 20 mg of protein was obtained per liter of cell culture. The molecular weight and molar extinction coefficient (ε280) of HS19.11_C_ were calculated to be 40 894 Da and 52,050 M^–1^ cm^–1^, respectively.^29^ Approximately 35 mg of protein were obtained per liter of cell culture. The enzyme Cj1435 was expressed and purified as previously described.^19^

#### Synthesis and Isolation of Compound **2a**

A 1.2 mL reaction containing 20 μM HS19.11_C_, 5.0 mM (*S*)-serinol phosphate, 5.0 mM methyl-β-d-glucuronic acid (**1a**), 5.0 mM ATP, and 5.0 mM MgCl_2_ in 50 mM NH_4_HCO_3_ (pH 8.0) was incubated at 25 °C for 18 h. The proteins were removed using 10 kDa MWCO Nanosep centrifugal filters. The reaction mixture was diluted to 15 mL with H_2_O and loaded onto a 5 mL HiTrap Q HP anion-exchange column connected to an NGC F10 chromatography system. The column was washed thoroughly with 50 mL of water. Compound **2a** was eluted from the column using a linear gradient of NH_4_HCO_3_ (0–250 mM) over a total elution volume of 100 mL, and the collected fractions were analyzed by ESI-MS. The fractions containing the desired product were pooled, lyophilized, and dissolved in D_2_O.

#### Synthesis and Isolation of Compound **3a**

Compound **3a** was obtained by dephosphorylation of compound **2a**. A 1.0 mL reaction containing 2.0 mM **2a**, 20 μM Cj1435, and 5.0 mM MgCl_2_ was incubated in 50 mM NH_4_HCO_3_, pH 8.0, for 18 h at 25 °C. Compound **3a** was purified using the same procedures as those used for compound **2a**. The uncharged compound **3a** eluted in the flow-through fractions, which were analyzed by ESI-MS. The appropriate fractions were combined, lyophilized, and then dissolved in D_2_O.

#### Synthesis and Isolation of Compound **4a**

A 1.2 mL reaction containing 20 μM HS19.11_N_, 5.0 mM UDP-GlcNAc, 5.0 mM compound **3a**, and 5.0 mM MgCl_2_ in 50 mM NH_4_HCO_3_, pH 8.0, was incubated for 18 h at 25 °C. The protein was removed using 10 kDa MWCO Nanosep centrifugal filters. The resulting filtrate was loaded onto a 5 mL HiTrap Q HP anion-exchange column connected to an NGC F10 chromatography system to remove the anionic contaminants and then washed with 10 column volumes of water. The fractions containing the desired product were pooled, lyophilized, and dissolved in D_2_O.

#### Synthesis and Isolation of Compounds **2b**–**4b**

Compounds **2b** through **4b** were synthesized and isolated using the same methods employed for the synthesis of compounds **2a**–**4a** with methyl-[^13^C-4]-d-glucuronic acid (**1b**) as the starting substrate. The isolated compounds **2b**, **3b**, and **4b**, were analyzed by ESI-MS and NMR spectroscopy.

#### Synthesis and Isolation of Compounds **6**–**8**

A 1.2 mL reaction containing 20 μM HS19.08_N_, 5.0 mM methyl-NAc-d-glucosamine (**5**), 5.0 mM UDP-GlcA, and 5.0 mM MgCl_2_ in 50 mM NH_4_HCO_3_, pH 8.0, was incubated for 18 h at 25 °C. Compound **6** was isolated from the resulting reaction mixture using the same methods employed for the isolation of **2a**. Compound **6** was converted to **7** in a 1.2 mL reaction mixture containing 20 μM HS19.11_C_, 2.0 mM compound **6**, 2.0 mM (*S*)-serinol phosphate, 2.0 mM ATP, and 5.0 mM MgCl_2_ in 50 mM NH_4_HCO_3_, pH 8.0. Compound **7** was isolated using the same methods employed for the isolation of compound **2a** after the reaction was incubated at 25 °C for 18 h. Compound **8** was obtained by dephosphorylation of **7** using Cj1435. A 1.2 mL reaction containing 2.0 mM compound **7**, 20 μM Cj1435, 5.0 mM MgCl_2_ in 50 mM NH_4_HCO_3_, pH 8.0, was incubated for 18 h at 25 °C. The uncharged compound **8** was isolated using the same methods employed for the isolation of compound **3a**. The isolated compounds **6**, **7**, and **8** were analyzed and verified by ESI-MS and NMR spectroscopy.

#### Synthesis and Isolation of Trimer **9**

A 1.2 mL reaction containing 20 μM HS19.08_N_, 20 μM HS19.11_N_, 20 μM 19.11_C_, 5.0 mM compound **3a**, 5.0 mM UDP-GlcNAc, 5.0 mM UDP-GlcA, 5.0 mM (*S*)-serinol phosphate, 5.0 mM ATP, and 5.0 mM MgCl_2_ in 50 mM NH_4_HCO_3_, pH 8.0, was incubated at 25 °C for 18 h. Trimer **9** was purified using the same methods employed for the isolation of compound **2a**. The isolated trimer was analyzed using ESI-MS and NMR spectroscopy.

#### Synthesis and Isolation of Pentamer **11**

A 0.5 mL reaction containing 20 μM Cj1435, 5.0 mM trimer **9**, and 5.0 mM MgCl_2_ in 50 mM NH_4_HCO_3_, pH 8.0, was incubated at 25 °C for 18 h. The enzyme Cj1435 was subsequently removed using a 10 kDa MWCO membrane filter. The resulting reaction mixture containing the dephosphorylated trimer **10** was then supplemented with 20 μM HS19.08_N_, 20 μM HS19.11_N_, 20 μM 19.11_C_, 5.0 mM UDP-GlcNAc, 5.0 mM UDP-GlcA, 5.0 mM (*S*)-serinol phosphate, 5.0 mM ATP, and 5.0 mM MgCl_2_ in 50 mM NH_4_HCO_3_, pH 8.0. The reaction was incubated at 25 °C for 18 h. The phosphorylated pentamer **11** was purified using the same methods employed for trimer **9**. The isolated pentamer **11** was analyzed using ESI-MS and NMR spectroscopy.

#### Synthesis and Isolation of Heptamer **13**

Heptamer **13** was obtained using the identical reaction and purification conditions used for the preparation of pentamer **11**, except that phosphorylated pentamer **11** was used as starting substrate instead of the phosphorylated trimer **9**.

#### Synthesis of Oligomeric Mixture

Two consecutive sets of enzymatic reactions were conducted to obtain a mixture of oligomeric products. First, a 1.2 mL reaction containing 20 μM HS19.08_N_, 20 μM HS19.11_N_, 20 μM HS19.11_C_, 20 μM Cj1435, 1.0 mM of the phosphorylated trimer **9**, 3.0 mM UDP-GlcNAc, 3.0 mM UDP-GlcA, 3.0 mM (*S*)-serinol phosphate, 5.0 mM ATP, and 5.0 mM MgCl_2_ in 50 mM NH_4_HCO_3_, pH 8.0, was incubated at 25 °C for 18 h. The enzymes were subsequently removed using a 10 kDa Nanosep centrifugal filter. The filtrate containing the oligomeric mixture (denoted here as **14-x**, where **x** is the number of disaccharide units added to the original trimeric starting material) was used to initiate a second reaction with the addition of another set of enzymes without Cj1435 (20 μM HS19.08_N_, 20 μM HS19.11_N_, and 20 μM HS19.11_C_) and substrates (1.0 mM UDP-GlcNAc, 1.0 mM UDP-GlcA, 1.0 mM (*S*)-serinol phosphate, 5.0 mM ATP, and 5.0 mM MgCl_2_) in 50 mM NH_4_HCO_3_, pH 8.0. The reaction mixture was incubated for 18 h at 25 °C. The oligomeric product mixture (denoted here as **15-x**) was purified using the same procedures described for compound **2a**, except that after protein removal, the reaction mixture was diluted to 30 mL to reduce the salt concentration before being loaded onto the anion-exchange column. The fractions containing the desired products, initially identified by ESI-MS, were pooled, lyophilized, and dissolved in D_2_O.

## Results and Discussion

The repeating disaccharide unit in the CPS from the HS:19 serotype of *C. jejuni* consists of the serinol amide of d-glucuronic acid and N-acetyl-d-glucosamine (Figure 1b). From the gene cluster that is responsible for the biosynthesis of this polysaccharide, the most likely pair of glycosyltransferases needed for the polymerization of these two monosaccharides are HS19.11 and HS19.08 (see Figure 2b). The AlphaFold2 predicted structures of these two enzymes are shown in Figure 4a and b, respectively.^30^ HS19.11 folds into a three-dimensional structure with three distinct domains. The N-terminal domain (colored purple) extends from residues 1–384, while the C-terminal domain (colored cyan) encompasses residues 512–832. These two domains are connected to one another by a 4-helix bundle (residues 385–511). The N-terminal domain (designated as HS19.11_N_) is a GT2 glycosyltransferase, while the C-terminal domain (labeled as HS19.11_C_) is an ATP-dependent ligase.^31,32^ For HS19.08, the N-terminal domain comprises residues 1–381 and is annotated as a GT2 glycosyltransferase.^33,34^ It is designated here as HS19.08_N_ and colored green in Figure 4b. Interestingly, neither of the GT2 glycosyltransferases (HS19.11_N_ and HS19.08_N_) is more than 20% identical in amino acid sequence to the GT2 glycosyltransferases functionally characterized from the HS:02 serotype (Cj1438_N_ and Cj1434).^24^ However, the ATP-dependent ligase (HS19.11_C_) is approximately 60% identical in sequence to the ligase (Cj1438_C_) previously characterized from the HS:02 serotype. The remaining enzyme of interest (HS19.09; the serinol-P phosphatase) is ∼66% identical in sequence to the previously characterized phosphatase from the HS:02 serotype (Cj1435). To elucidate the biochemical transformations needed to synthesize the undecorated CPS from the HS:19 serotype of *C. jeuni*, we purified HS19.11_N_, HS19.11_C_, and HS19.08_N_ and utilized the previously characterized Cj1435 as the potential phosphatase.

**Figure 4:**
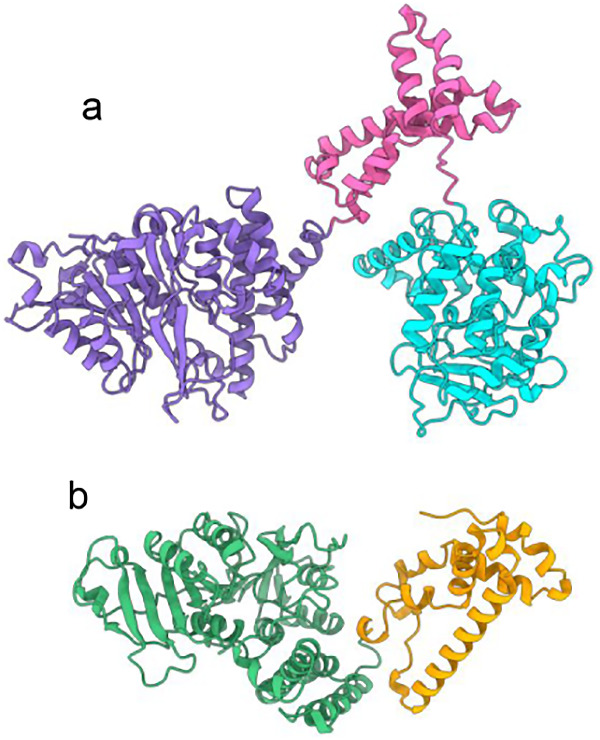
Alphafold2 predicted structures of HS19.11 (a) and HS19.08 (b). Additional details are provided in the text.

The proposed routes for the biochemical characterization of the two GT2 glycosyltransferases needed to synthesize the undecorated CPS from the HS:19 serotype of *C. jejuni* are provided in Figure 5. In the upper pathway the β-methyl glycoside of d-GlcA (**1a**) is amidated with (*S*)-serinol-P using ATP and HS19.11_C_ to form compound **2a**. In the second step the phosphate moiety is removed by the catalytic activity of HS19.09 and in the last step disaccharide **4a** is formed using **3a**, UDP-GlcNAc, and either HS19.11_N_ or HS19.08_N_ as the glycosyltransferase. In the first step, incubation of **1a**, MgATP, (*S*)-serinol-P and HS19.11_C_ resulted in the formation of ADP and product **2a**, which was subsequently isolated by anion exchange chromatography. The proposed structure for **2a** was supported by ESI-MS with an observed *m*/*z* of 360.07 for the [M–H^+^]^−^ anion (calculated *m*/*z* = 360.07). In the second step the phosphate of **2a** was hydrolyzed using Cj1435 (as a functional substitute for HS19.09) and the progress of the reaction followed by ^31^P NMR spectroscopy. Product formation was supported by ESI-MS with an *m*/*z* of 282.12 for the [M+H^+^]^+^ cation of **3a** (calculated *m*/*z* = 282.12). In the last step disaccharide **4a** was made by incubation of **3a**, UDP-GlcNAc, and HS19.11_N_. No reaction was observed upon substitution of HS19.08_N_ as the glycosyltransferase. The structure of disaccharide **4a** was supported by ESI-MS with an *m*/*z* of 485.20 for the [M+H^+^]^+^ cation (calculated *m*/*z* = 485.20). The ^1^H NMR spectrum of disaccharide **4a** highlighting the two anomeric hydrogens is illustrated in Figure 6a. The two anomeric hydrogens are clearly separated from one another as doublets at 4.41 and 4.49 ppm, originating from the GlcA and GlcNAc moieties of the disaccharide, respectively.

**Figure 5:**
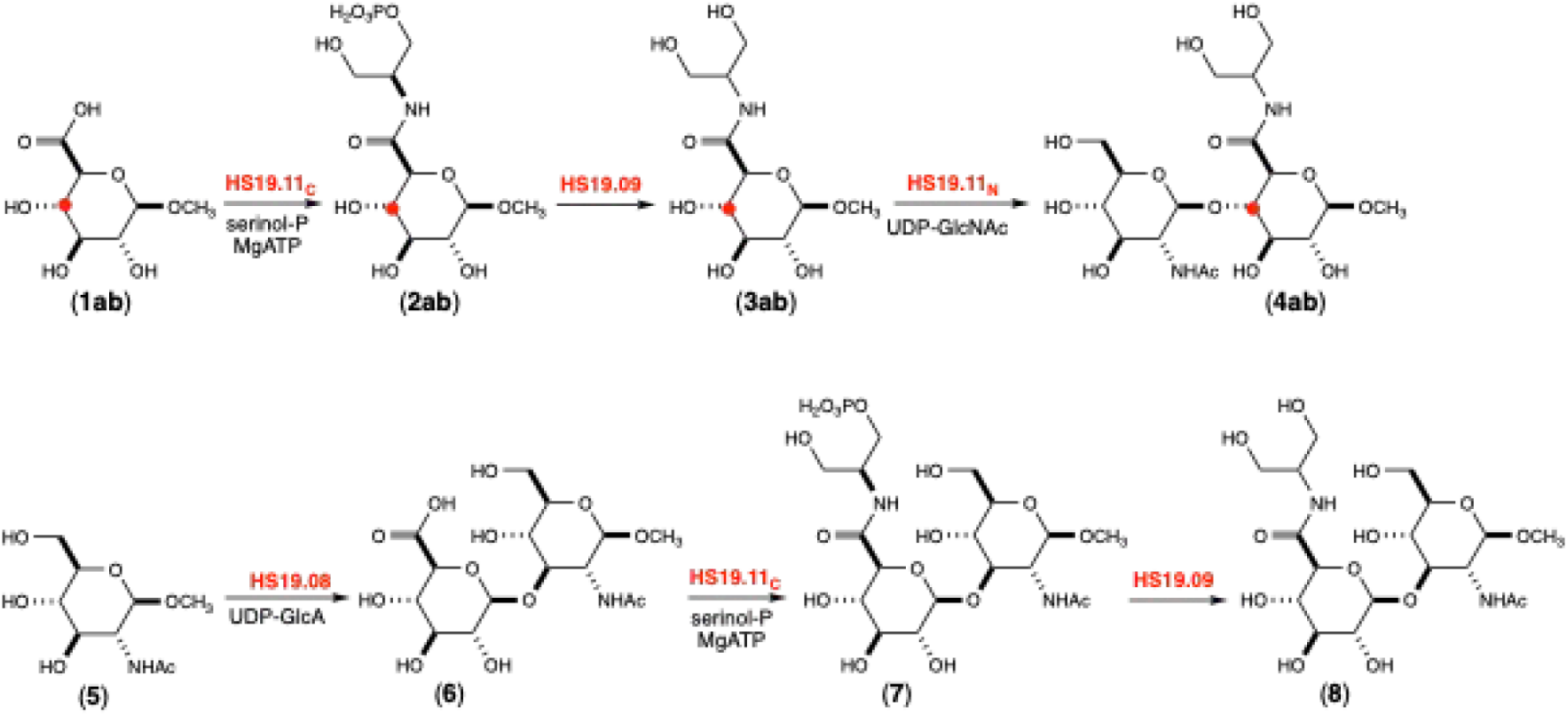
Proposed enzymatic transformations for the synthesis of the undecorated capsular polysaccharide from the HS:19 serotype of *C. jejuni*. In the upper panel those compounds designated as “**a**” do not contain a ^13^C-label at C4, whereas those designated as “**b**” contain a ^13^C-label at C4 of the GlcA moiety.

**Figure 6:**
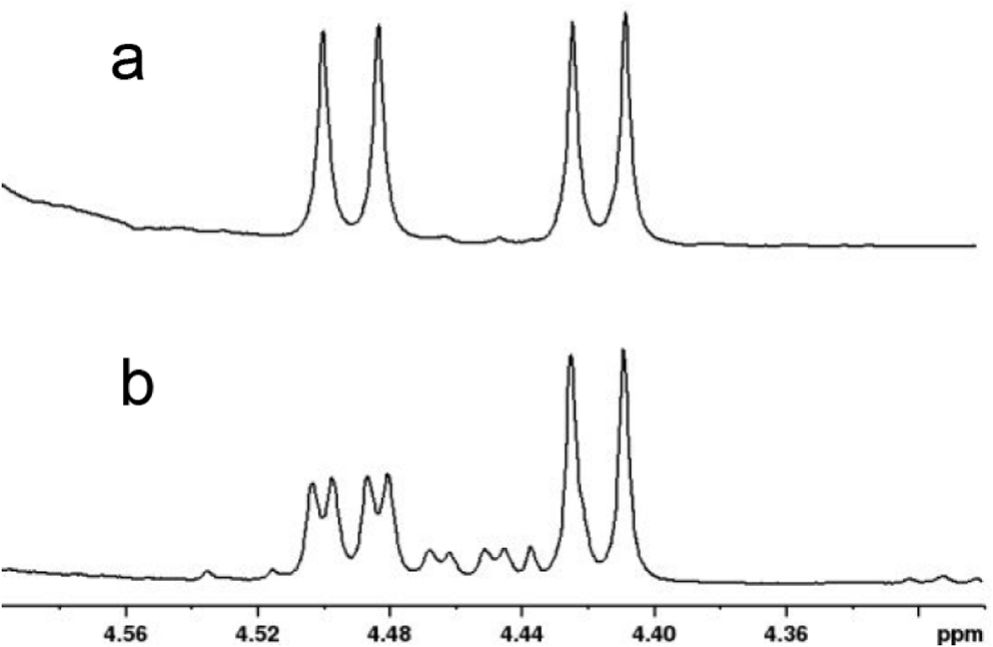
Portion of the ^1^H NMR spectrum of compound **4a** (shown in panel a) and compound **4b** (shown in panel b). In panel a, the doublet at 4.41 ppm originates from the anomeric hydrogen of the GlcA moiety of disaccharide **4a**, while the doublet at 4.49 ppm originates from the anomeric hydrogen of the GlcNAc moiety. In panel b, the doublet at 4.41 ppm originates from the anomeric hydrogen of the GlcA moiety of disaccharide **4b**, while the doublet of doublets centered at 4.49 ppm originates from the anomeric hydrogen of the GlcNAc moiety, clearly showing spin coupling with the ^13^C-label at C4 of the GlcA moiety. The doublet of doublets centered at 4.46 ppm originates from a contamination of compound **4b** where the positioning of -OMe group is α instead of β.

The specific glycosidic linkage for disaccharide **4a** was confirmed via the enzymatic synthesis of **4b** starting with the^13^C-labeled methyl-glycoside of d-GlcA (**1b**) and progressing through the formation of compounds **2b** and **3b**. The structure of **2b** was supported by ESI-MS with an observed *m*/*z* of 361.07 for the [M–H^+^]^−^ anion (calculated *m*/*z* = 361.08). Compound **2b** was dephosphorylated using Cj1435 and then coupled to GlcNAc using HS19.11_N_ with UDP-GlcNAc. The structure was supported via ESI-MS showing an *m*/*z* of 486.20 for the [M+H^+^]^+^ cation (calculated *m*/*z* = 486.20). The partial ^1^H NMR spectrum of disaccharide **4b** clearly shows the doublet of doublets for the anomeric hydrogen of the GlcNAc moiety at ∼4.49 ppm indicating that the conjugation of the two sugars occurs via a linkage at C4 of the GlcA moiety, as expected from the previously determined structure of the CPS isolated from *C. jejuni* (Figure 6b).^26,27^

The lower pathway shown in Figure 5 illustrates the proposed series of reactions for the synthesis of the inverted disaccharide **8** and confirmation of the catalytic activity of the glycosyltransferase that adds the d-GlcA moiety to the nonreducing end of the growing polysaccharide chain. In the first step the β-methyl-glycoside of GlcNAc (**5**) was incubated with UDP-GlcA and HS19.08_N_ and the reaction following by the formation of UDP via anion exchange chromatography. Disaccharide **6** was isolated by anion exchange chromatography and the proposed structure supported by ESI-MS with an observed *m*/*z* for the [M–H^+^]^−^ anion of 410.13 (calculated *m*/*z* = 410.13). No reaction was observed when HS19.11_N_ was used as the glycosyltransferase.

Disaccharide **6** was subsequently used as the substrate for the ATP-dependent ligase HS19.11_C_ using (*S*)-serinol-P and ATP as the other two substrates. Disaccharide **7** was isolated by anion exchange chromatography and the structure supported by ESI-MS with an observed *m*/*z* for the [M–H^+^]^−^ anion of 563.15 (calculated *m*/*z* of 563.15). The NMR spectra for compounds **2a**, **5** and **7** are shown in Figures S2–S8. The condensation of the GlcA and GlcNAc moieties to one another in compound **7** is confirmed to be at C3 of the GlcNAc moiety via the 8.6 ppm change in the ^13^C-chemical shift for C3 in compound **7** (82.6 ppm) relative to that found in compound **5** (74.0 ppm). This perturbation has been denoted previously as the glycosylation shift.^35^ In the final step, disaccharide **8** was formed via the hydrolysis of the phosphate moiety of **7** using the phosphatase Cj1435. The structure of disaccharide **8** was supported by ESI-MS for the [M+H^+^]^+^ cation of 485.20 (calculated *m*/*z* of 485.20), which is exactly the same as that observed for the disaccharide of the inverse sequence, **4a**.

These two series of reactions demonstrate that HS19.08 is used to transfer d-GlcA from UDP-GlcA to C3 of GlcNAc at the reducing end of the growing capsular polysaccharide. In the subsequent step, the C-terminal domain of HS19.11 catalyzes ATP-dependent amide bond formation between (*S*)-serinol-phosphate and the carboxylate of the GlcA moiety. The phosphate is removed via hydrolysis by HS19.09 and then the N-terminal domain of HS19.11 transfers GlcNAc from UDP-GlcNAc to C4 of the amidated GlcA moiety. Like the observed reactions catalyzed during the biosynthesis of the CPS in the HS:02 serotype, the reaction catalyzed by HS19.11 must use the unphosphorylated serinol glucuronamide as the sugar acceptor.^32^

### Enzymatic Synthesis of Oligomers of Defined Length

The strategy for the synthesis of oligomers containing 3, 5, and 7 monomeric units is illustrated in Figure 7. The synthesis of trimer **9** begins with the incubation of monomer **3a** and one equivalent each of UDP-GlcA, UDP-GlcNAc, (*S*)-serinol-P, and MgATP in the presence of HS19.11_N_, HS19.11_C_, and HS19.08_N_. The series of reactions must stop at the phosphorylated trimer **9** because the phosphate must be removed before any subsequent reactions can occur. Trimer **9** was isolated by anion exchange chromatography and then the phosphatase, Cj1435, was added to stoichiometrically convert trimer **9** to trimer **10**. The phosphatase was quantitatively removed by filtration and then another round of synthesis proceeded as before resulting in the synthesis and isolation of pentamer **11**. The two sets of reactions were repeated one more time to enable the isolation of heptamer **13** via the intermediate formation of heptamer **12**. Confirmation of the proposed structures for oligomers **9**, **11**, and **13** was determined by ESI-MS and ^1^H NMR spectroscopy. The ESI-MS for trimer-**9** exhibited an *m*/*z* of 812.13 for the [M–H^+^]^−^ anion of this phosphorylated species (calculated *m*/*z* of 812.13). For pentamer **11** and heptamer **13**, the dianion (*z* = 2) was the most prominent species observed in the mass spectrum, where pentamer-**11** exhibited an *m*/*z* of 631.70 for the [M-2H^+^]^2–^ dianion (calculated *m*/*z* = 631.69). For heptamer-**13** the dianion (*z* = 2) was observed at an *m*/*z* = 857.78 for the [M-2H^+^]^2–^ dianion (calculated *m*/*z* = 857.78). The mass spectral data for oligomers **9**, **11**, and **13** are graphically presented in Figures S9 and S10, and summarized in Table S1. The ^1^H NMR spectra for compounds **4a**, **9**, **11**, and **13** are shown in Figure S11.

**Figure 7:**
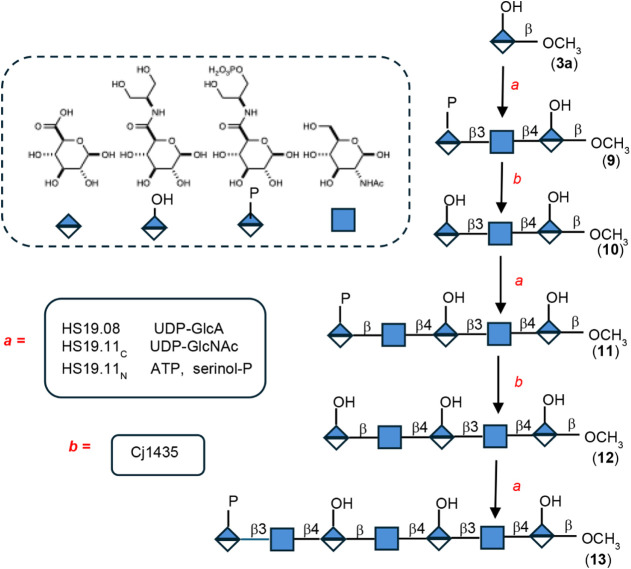
Strategy for the enzymatic synthesis of oligomers **9**, **11**, and **13**.

### Enzymatic Synthesis of Longer Unfractionated Oligomers

The strategy for the synthesis of longer oligomers of the capsular polysaccharide from the HS:19 serotype of *C. jejuni* is presented in Figure 8. Here we incubated the phosphorylated trimer **9** with three equivalents of GlcA, GlcNAc, (*S*)-serinol-phosphate, MgATP, and the enzymes HS19.11_N_, HS19.11_C_, HS19.08_N_, and Cj1435. This procedure was predicted to generate a mixture of oligomers of different lengths, whose overall composition will depend on the collective processivity of the four enzymes required for extension of the growing oligosaccharide. The average length is estimated to be 9 carbohydrate units. In the second step, the enzymes were first removed by filtration and then the ragged ends of the oligomeric mixture were extended and subsequently terminated with a phosphorylated serinol amide of GlcA by adding one equivalent each of GlcA, GlcNAc, (*S*)-serinol-phosphate, and MgATP, along with HS1911_N_, HS19.11_C_, and HS19.08_N_. The absence of the phosphatase ensures that all the oligosaccharides will terminate at the nonreducing end with a phosphorylated serinol amide of GlcA. The average length of the oligosaccharide mixture was predicted to contain 11 carbohydrate units. These oligosaccharides were collectively isolated via anion exchange chromatography. Shown in Figure 9 is the ESI-MS of the isolated mixture, which clearly shows the presence of the nonamer **15–2**, undecamer **15–3**, tridecamer **15–4**, and pentadecamer **15–5**. The predominant species observed in the ESI-MS was the [M–H^+^+2Cl^–^]^3–^ anion with smaller amounts of [M–H+Cl^–^]^2–^ anion. Unfortunately, we were unable to quantitatively determine the specific ratios of these oligosaccharides with one another. The expanded ESI-MS for each anion is presented in Figures S12 and S13, and Table S2.

**Figure 8:**
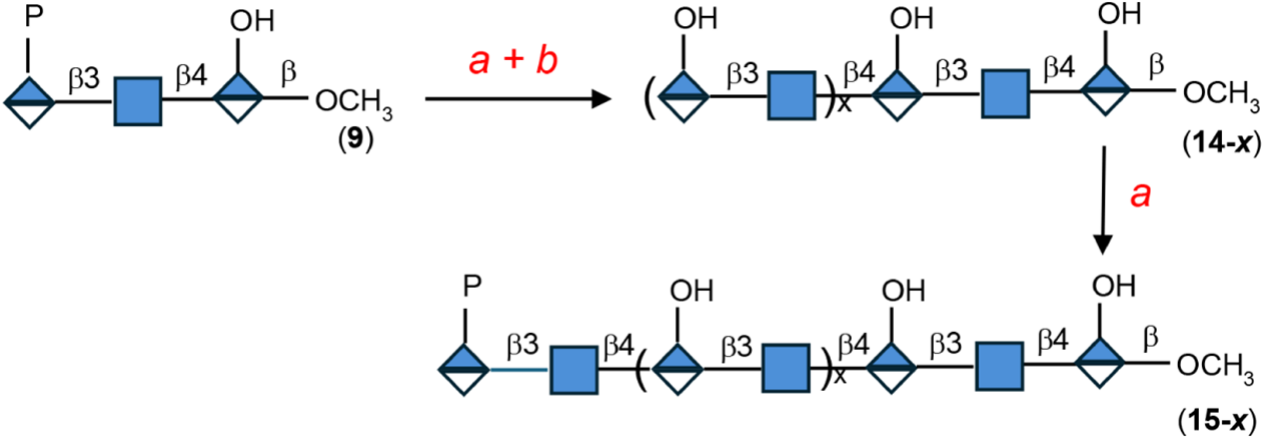
Strategy for the enzymatic synthesis of longer oligomers of the capsular polysaccharide of the HS:19 serotype of *C. jejuni*. The cartoon representation of each sugar unit and the reaction conditions denoted by **a** and **b** are the same as that defined in Figure 7.

**Figure 9:**
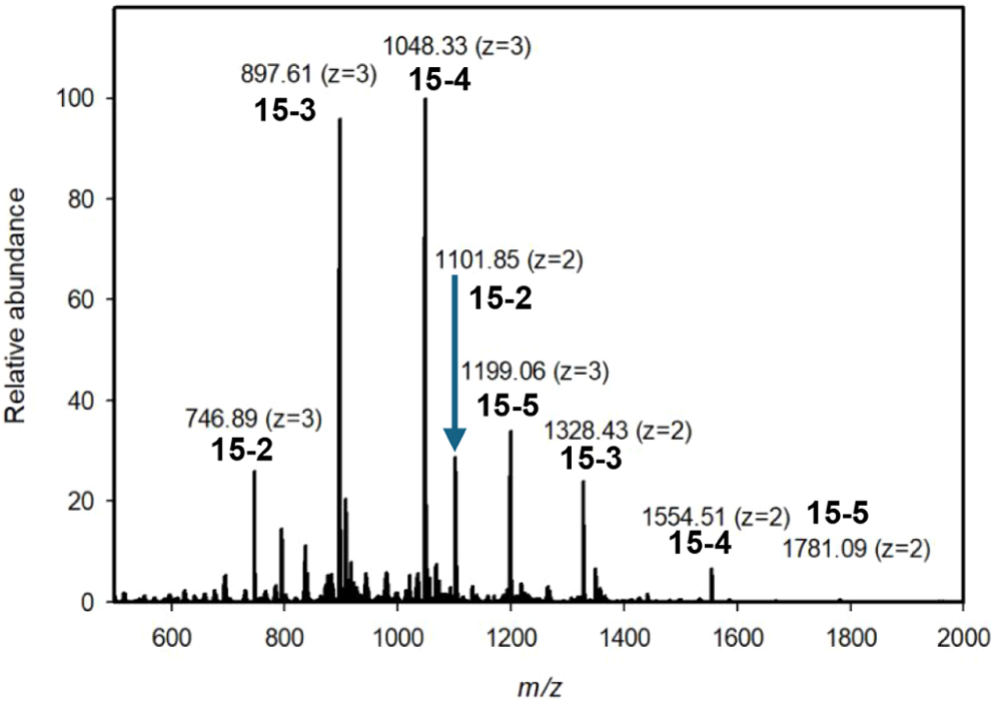
ESI-MS of the unfractionated product mixture containing nonamer (**15–2**), undecamer (**15–3**), tridecamer (**15–4**), and pentadecamer (**15–5**). The species with *z* = 3 correspond to the product after the loss of one proton and addition of two chloride ions, [M–H^+^+2Cl^–^]^3–^. The species with *z* = 2 correspond to the product after loss of one proton and addition of 1 chloride ion, [M–H^+^+Cl^–^]^2–^.

## Conclusions

The repeating disaccharide unit in the undecorated capsular polysaccharide from the HS:19 serotype of the human pathogen *C. jejuni* consists of N-acetyl-d-glucosamine and the serinol amide of d-glucuronic acid.^26,27^ We have now demonstrated that the N-terminal domain of HS19.08 catalyzes the transfer of d-glucuronic acid from UDP-GlcA to C3 of the GlcNAc moiety at the nonreducing end of the growing polysaccharide. The carboxylate of the added GlcA is then amidated by the ATP-dependent ligase activity of the C-terminal domain of HS19.11 using (*S*)-serinol-phosphate. The phosphorylated serinol amide is subsequently dephosphorylated by the catalytic activity of HS19.09. In the final step in the series, the N-terminal domain of HS19.11 catalyzes the transfer of NAc-d-glucosamine from UDP-GlcNAc to C4 of the amidated glucuronic acid. These four enzymatic steps are sequentially repeated to polymerize the CPS, and the overall biosynthetic pathway is graphically summarized in Figure 10. We have demonstrated that the minimal acceptor substrate for these two polymerizing glycosyltransferases is a single O-methyl glycoside. We have exploited this series of reactions to enzymatically synthesize and isolate defined oligomers containing 2, 3, 5, 7, and 9 monomeric units. We have also developed procedures for the synthesis of oligomeric mixtures ranging from 9 to 15 monomeric units. It is not clear what reaction conditions limit the size of the polysaccharide in the cell, and we have not identified the specific set of enzymes and/or reaction conditions that enable the first and/or second sugar to be added to the poly-KDO linker. However, it is highly likely that one or both reactions will be catalyzed by HS19.13 since this enzyme is the only uncharacterized glycosyltransferase from this gene cluster. In addition, the enzymatic details for decorating the capsular polysaccharide with a methylated phosphoramidate group at C5 of the GlcNAc moiety are also currently unclear.

**Figure 10:**
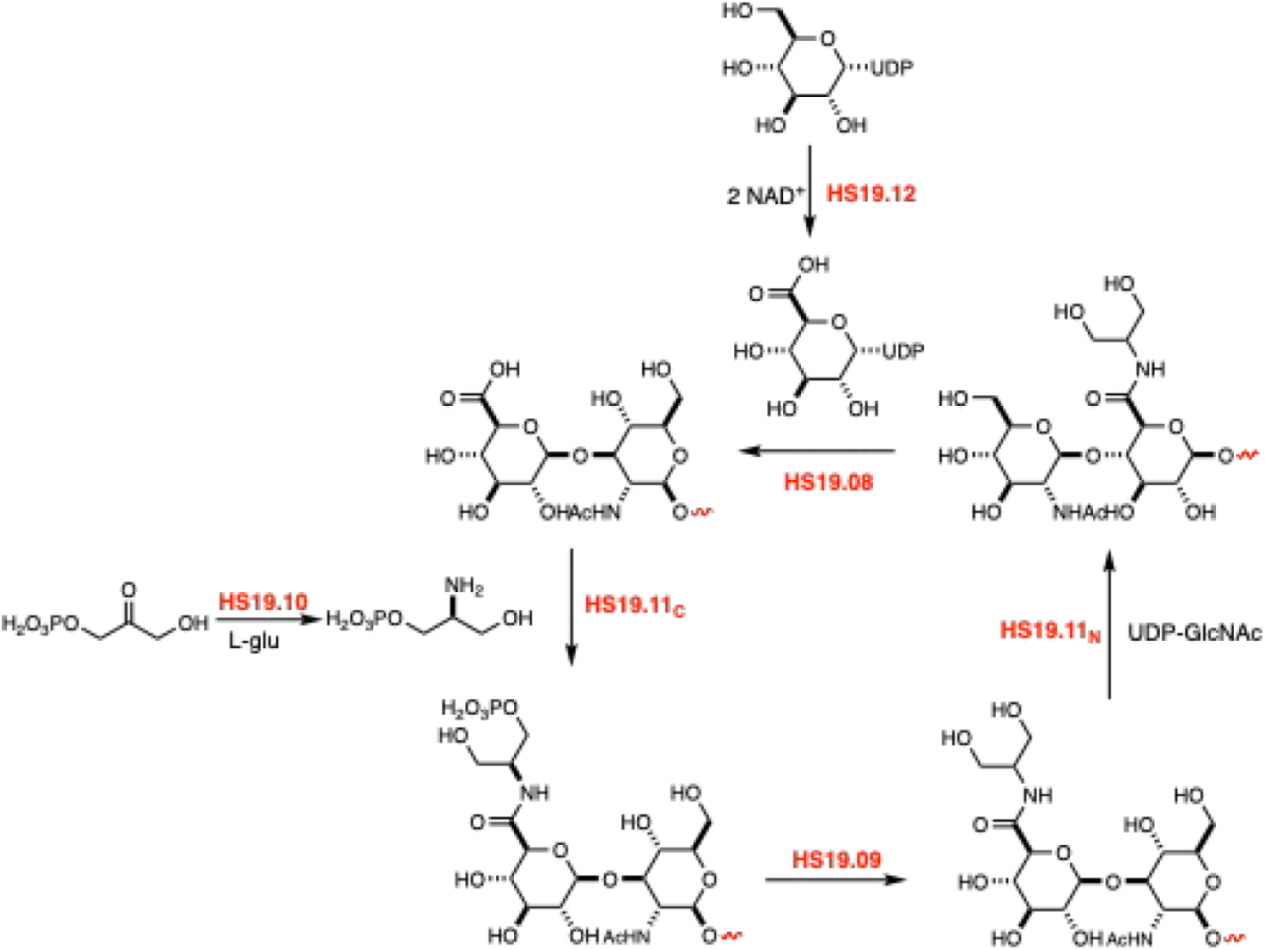
Biosynthetic pathway for the elongation of the undecorated capsular polysaccharide of the HS:19 serotype of *C. jejuni*. In this scheme we have shown the reactions catalyzed at the nonreducing end of the polymer by each enzyme in the cycle. Since two of these reactions (catalyzed by HS19.08 and HS19.11_N_) are making the polymer longer, we have arbitrarily removed one sugar moiety from the reducing end of the polymer for those steps to make the figure manageable.

## Supplementary Material



## Notes

Amino acid sequences of the purified proteins, and ESI-MS and NMR spectra of enzyme-catalyzed reaction products (PDF)
