# Supplementary material for: Elucidation of the Pathway for the Biosynthesis of the Undecorated Capsular Polysaccharide from the HS:19 Serotype of the Human Pathogen Campylobacter jejuni
Source: Biochemistry. 2026 Jun 4;65(12):1978–87. doi: 10.1021/acs.biochem.6c00264 (PMC13276843; doi:10.1021/acs.biochem.6c00264)
Supplement: Supplementary file 1 [file bi6c00264_si_001.pdf]

Supporting Information

Elucidation of the Pathway for the Biosynthesis of the Undecorated Capsular Polysaccharide from the HS:19 Serotype of the Human Pathogen *Campylobacter jejuni*

Dao Feng Xiang, Tamari Narindoshvili, and Frank M. Raushel*

Department of Chemistry, Texas A&M University, College Station, Texas, United States 77842

*Contact Information

email: raushel@tamu.edu

(a) HS19.08$_N$ (1-381 amino acids; UniProt id: Q5M6M6)

MGSSHHHHHHSSENLYFQGHMKTVGVVIPIYNVEKYLRECLDSVVNQTYKNLQVVLVNDGSTDENSLNIA
KEYTLKDERFILFDKENGGQSTARNVGIEFFSKEYDFKNITQELKENSLVEFKLDNEDNPYNIYKIYKSS
NFFKNKDELLNFKAPDIDYIIFLDSDDYWELNCIEECVPRMDGVEVVWFDYNKIYEKDCLEKKDEWTWFN
CYNMGIKKDIIISDEWLDKYCNIQTFAFVWSGMIAFNYLSNQKIKFLDYIFHQDVYFGFMVFFKSNKISL
LNKKIINYRIRSNATTLRQGKIGEQIILPKYLDFLSKFYNKNEDAKKYYSLFSWSKMVEKAIEDSFYDEK
NIIINYFLPSLCMQLFQKDINKNLDPLNIGIYINFSKVIFKMFRLKHQNII

(b) HS19.11$_N$ (1-384 amino acids; UniProt id: Q5M6M2)

MGSSHHHHHHSSENLYFQGHMKTVGVVIPIYNVEKYLRECLDSVVNQTYKNLQVVLVNDGSTDENSLNIA
KEYTLKDERFILFDKENGGQSTARNVGIEFFSKEYDFKNITQELKENSLVEFKLDNEDNPYNIYKIYKSS
NFFKNKDELLNFKAPDIDYIIFLDSDDYWELNCIEECVPRMDGVEVVWFDNKAFDYEIKTIYPTSKTFME
CFNYNIKNKQINGNTWFDECRKNNITSIWIAVMEMIDFAYLKTLKLKFLDGVLYEDNLFGTLLFLNVKKL
YVLDKKLYNNRIRANSTMCHDNNLSFENLAPFFRILSNDFLDPYDAREYIKLHSWTCMTFVLLLMYVNKF
KNKENLEKIRFFLFSYKDILFENIKLNQDPWAIKDKIDIINFFVNNKFKDNKYQ

(C) HS19.11$_C$ (513-832 amino acids; UniProt id: Q5M6M2)

MGSSHHHHHHSSENLYFQGHECNLEEDEIFFKERHKAIFNYIPDFKHPQTFNEKLVFRMLYDRSPLYTFL
ADKLKMRIFIQQILSQFDESNIFDNNSVLFQDIDKIQDKILNTNICEYLPKLYAIYDDIYDIDFDILPES
FVLKTNHDCGGYVIVEDKIKFLRDIDLFSSSMQKLHNHLHSNYYYLSREWHYKDIKPKIFAEELLIDKNG
KLADTYKFHIFDHKNLNNNYIQVTTDRFNNYQRFIMDSNWNIAPFNFTYEVSKDKLPNRPSEFEKMFEIS
LKLSKMFDYVRVDLYCIDNRIYIGELTFTHGAAGEKLNPNCWDKKLGKLWNIRKLSDVAK

Figure S1. Amino acid sequences for the three proteins used in this investigation. (a) Amino acid sequence for HS19.08$_N$. The polyhistidine tag at the N-terminal end is shown in red. (b) Amino acid sequence for HS19.11$_N$. The polyhistidine tag at the N-terminal end is shown in red. (c) Amino acid sequence for HS19.11$_C$. The polyhistidine tag at the N-terminal end is shown in red.

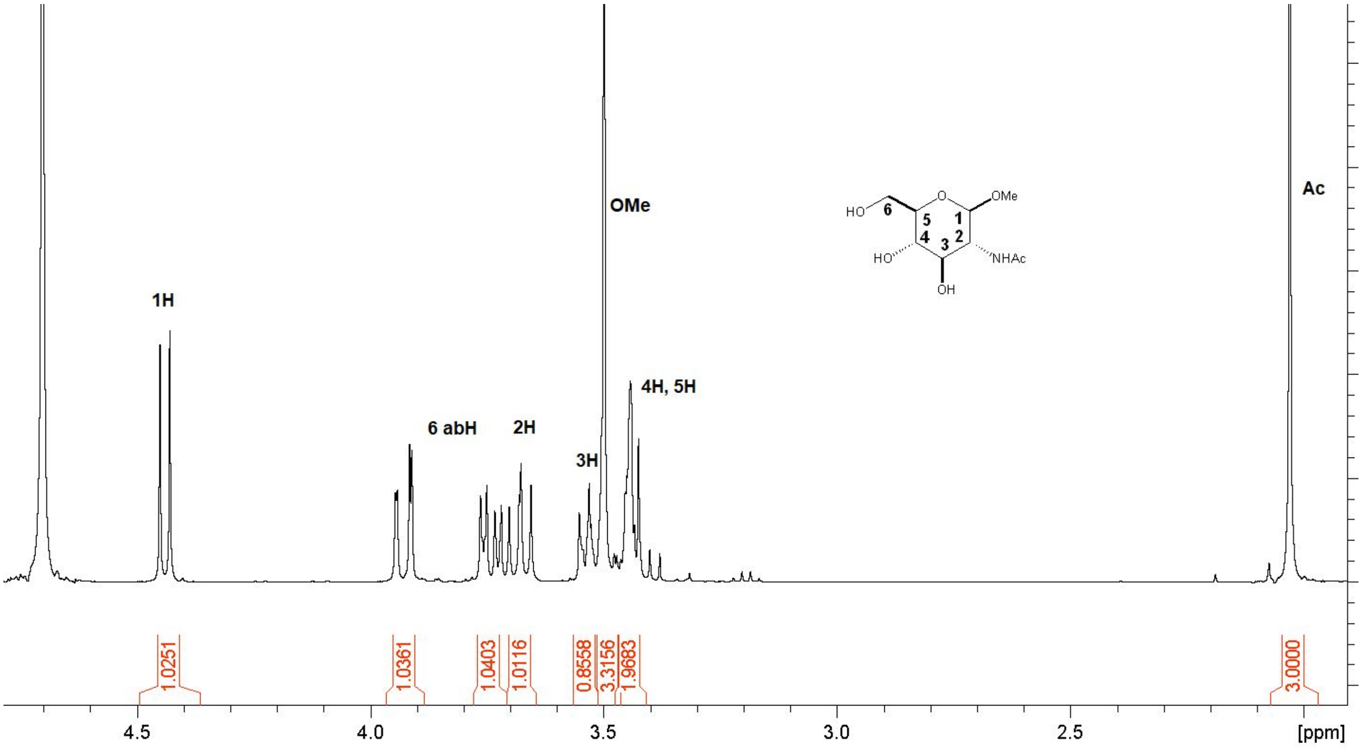

Figure S2. ^{1}H NMR spectrum of compound **5** in D_2O (400 MHz).

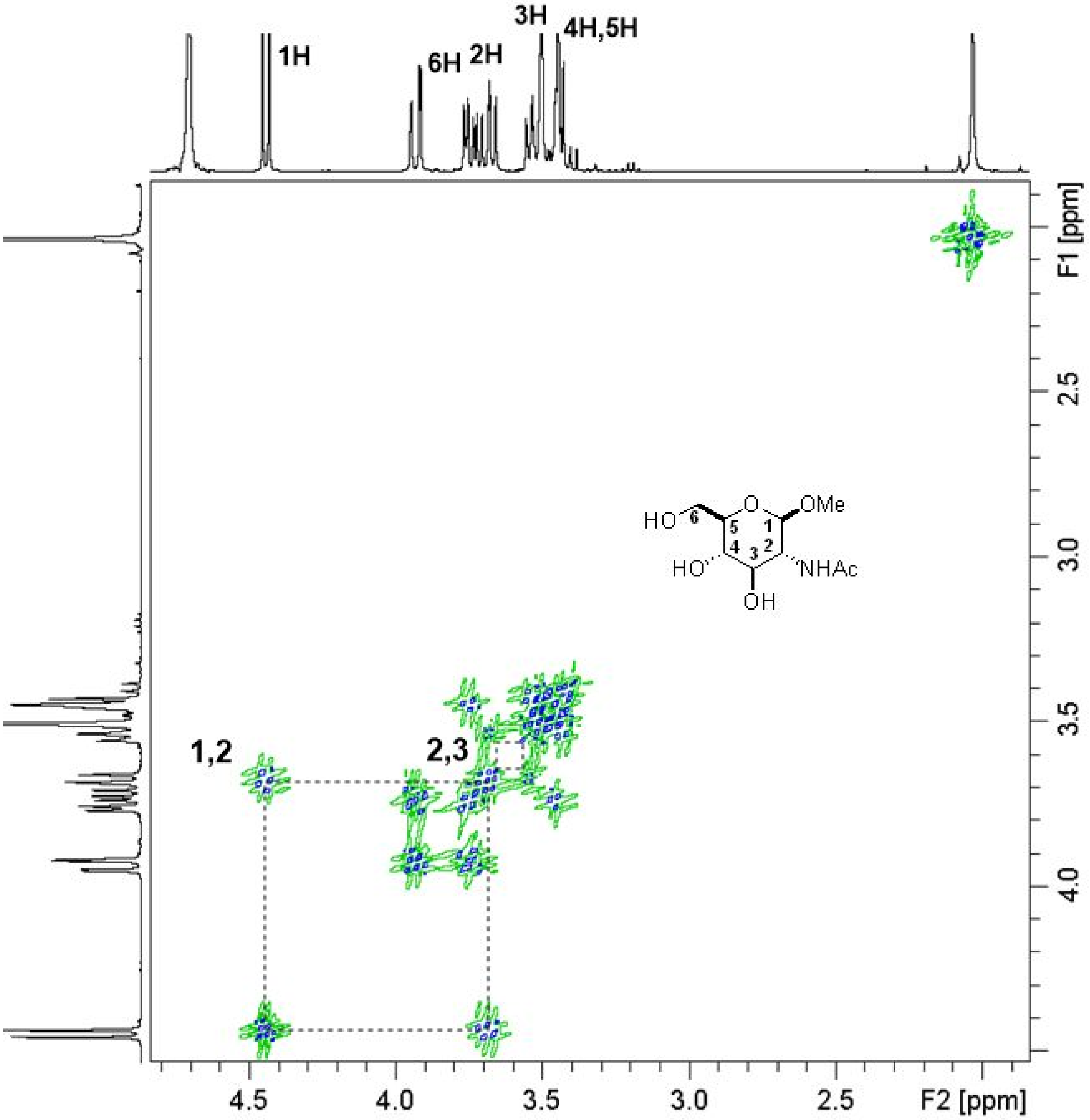

Figure S3. COSY 2D spectrum of compound **5** in D_2O. Starting from the anomeric proton (H-1) at 4.44 ppm, a cross-peak identifies H-2 at 3.66 ppm. From H-2, a cross-peak with H-3 is observed at 3.53 ppm. H-3 shows correlations with H-4 and H4 with H-5, both appear in the region of 3.45–3.42 ppm. Finally, H-6 is identified at 3.83 ppm through its coupling with H-5.

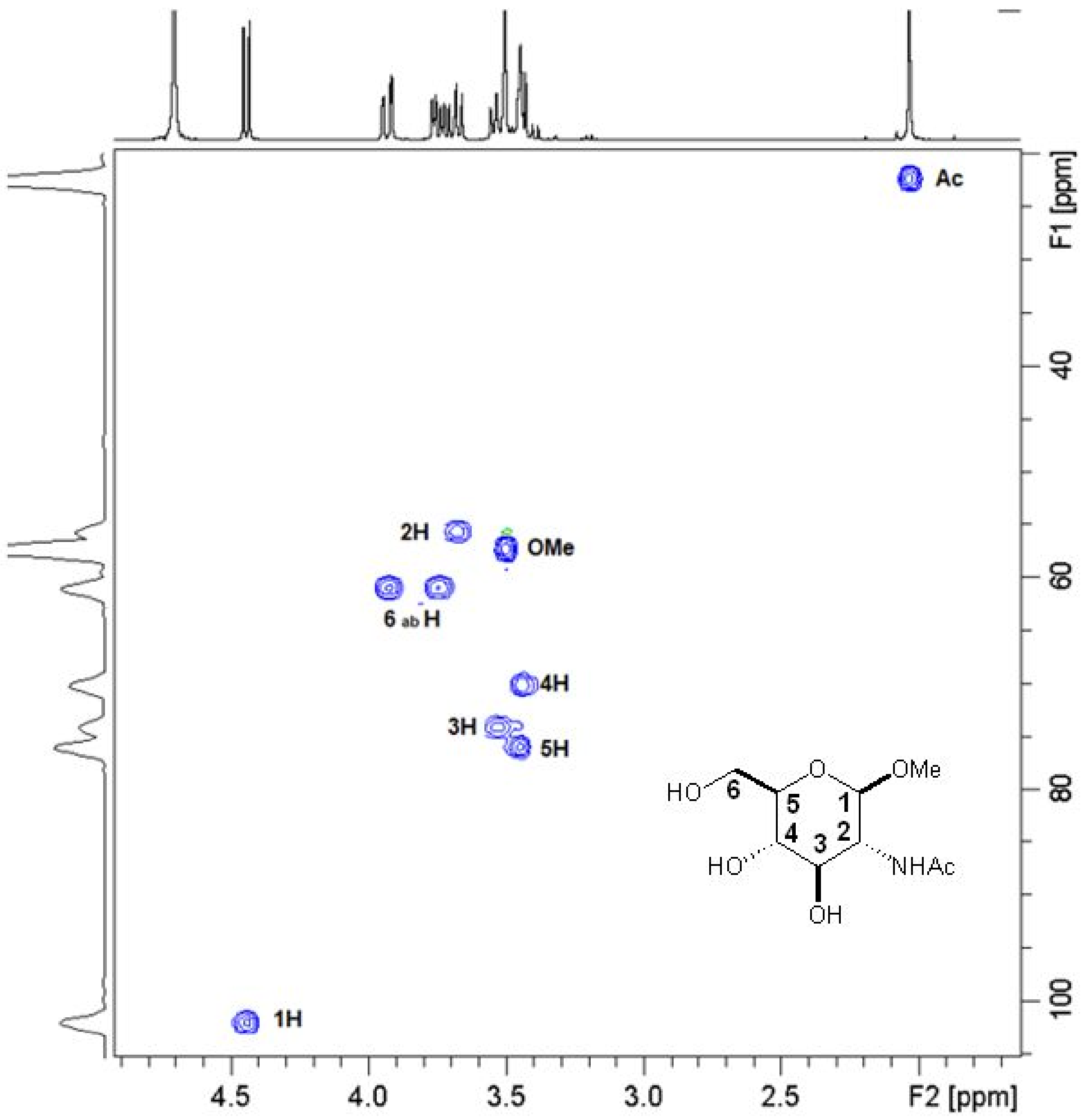

Figure S4. HSQC 2D spectrum of compound **5** in D_2O. In compound **5**, the ^{13}C NMR signals corresponding to C-3, C-4, and C-6 were observed at δ 74.0, 70.1, and 60.9 ppm, respectively.

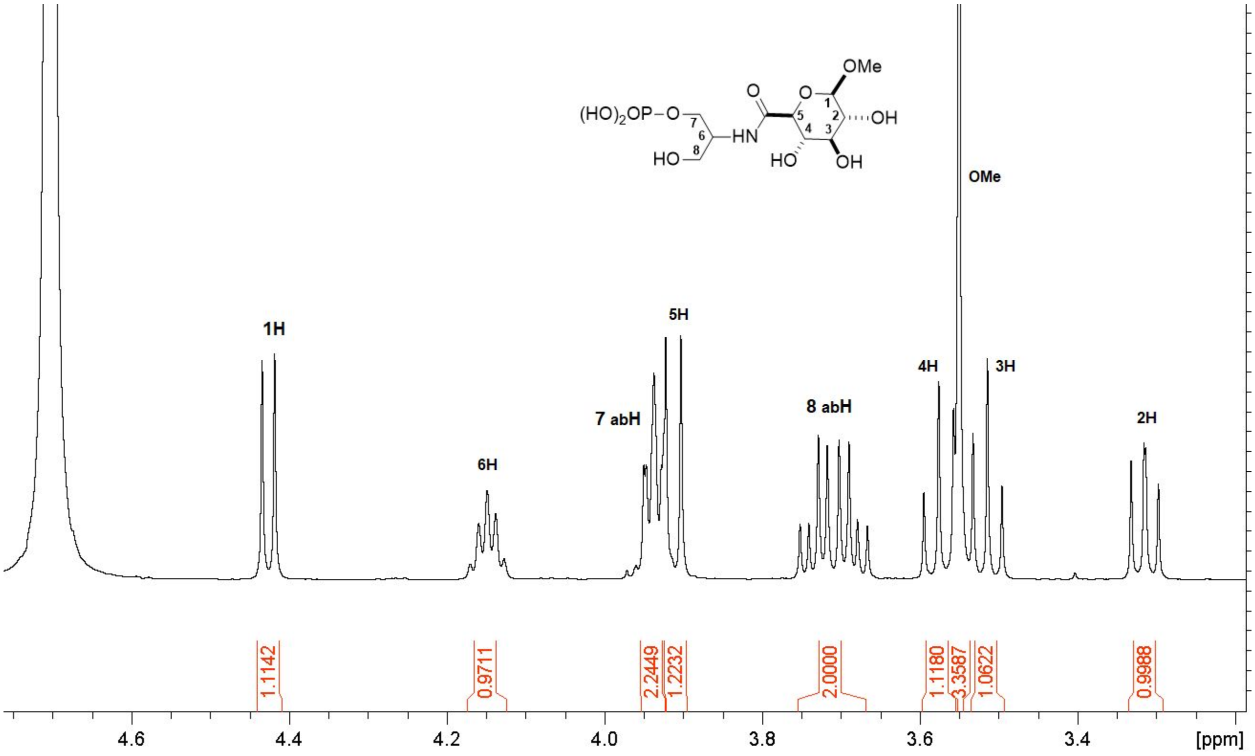

Figure S5. ^{1}H NMR spectrum of compound **2a** in D_2O (400 MHz).

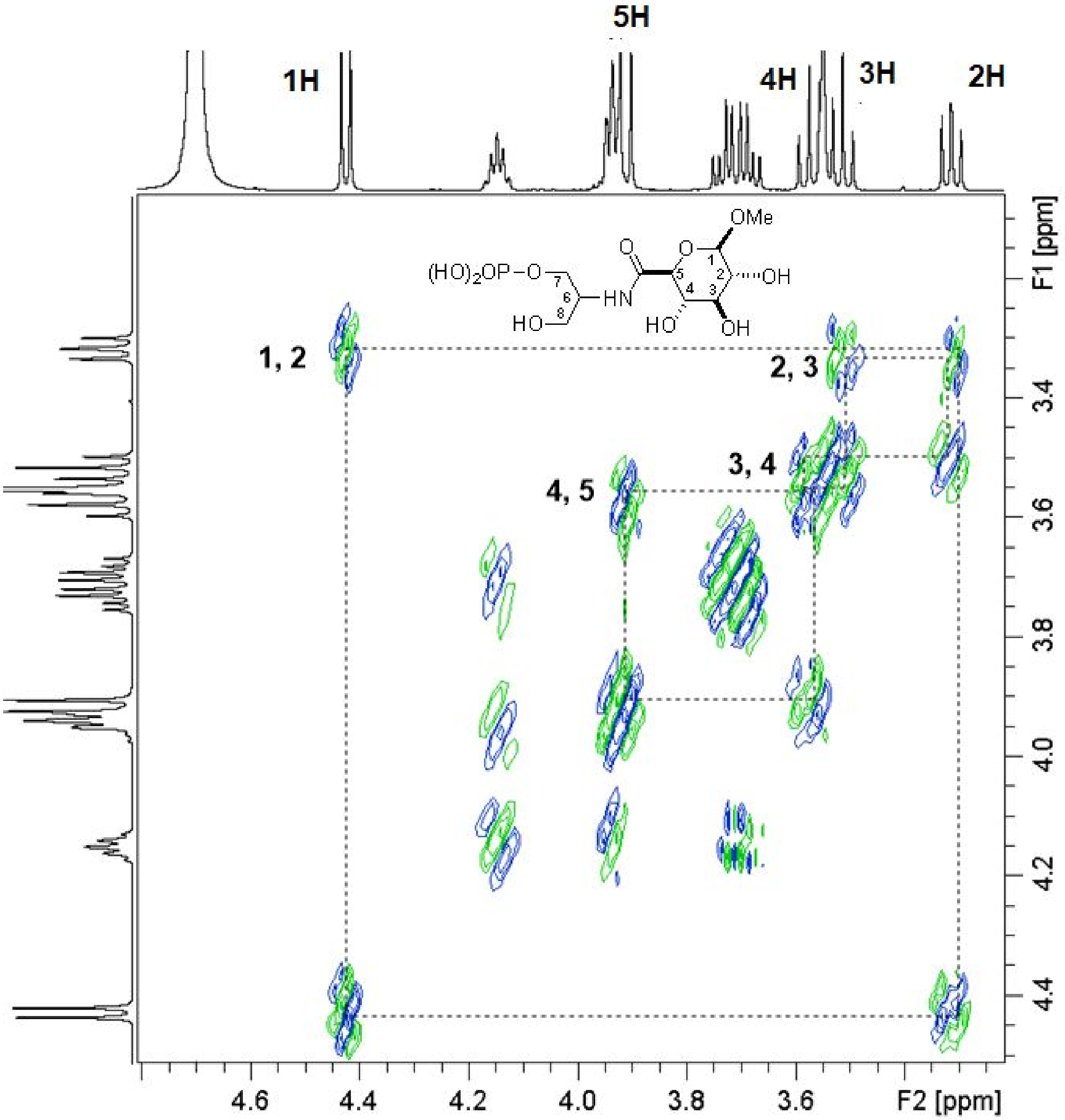

Figure S6. COSY 2D spectrum of **2a** in D_2O. Starting from the anomeric proton (H-1) at 4.42 ppm, a cross-peak identifies H-2 at 3.31 ppm. From H-2, a cross-peak with H-3 is observed at 3.51 ppm. H-3 shows correlations with H-4 at 3.57 ppm and H-4 with H-5 at 3.91 ppm.

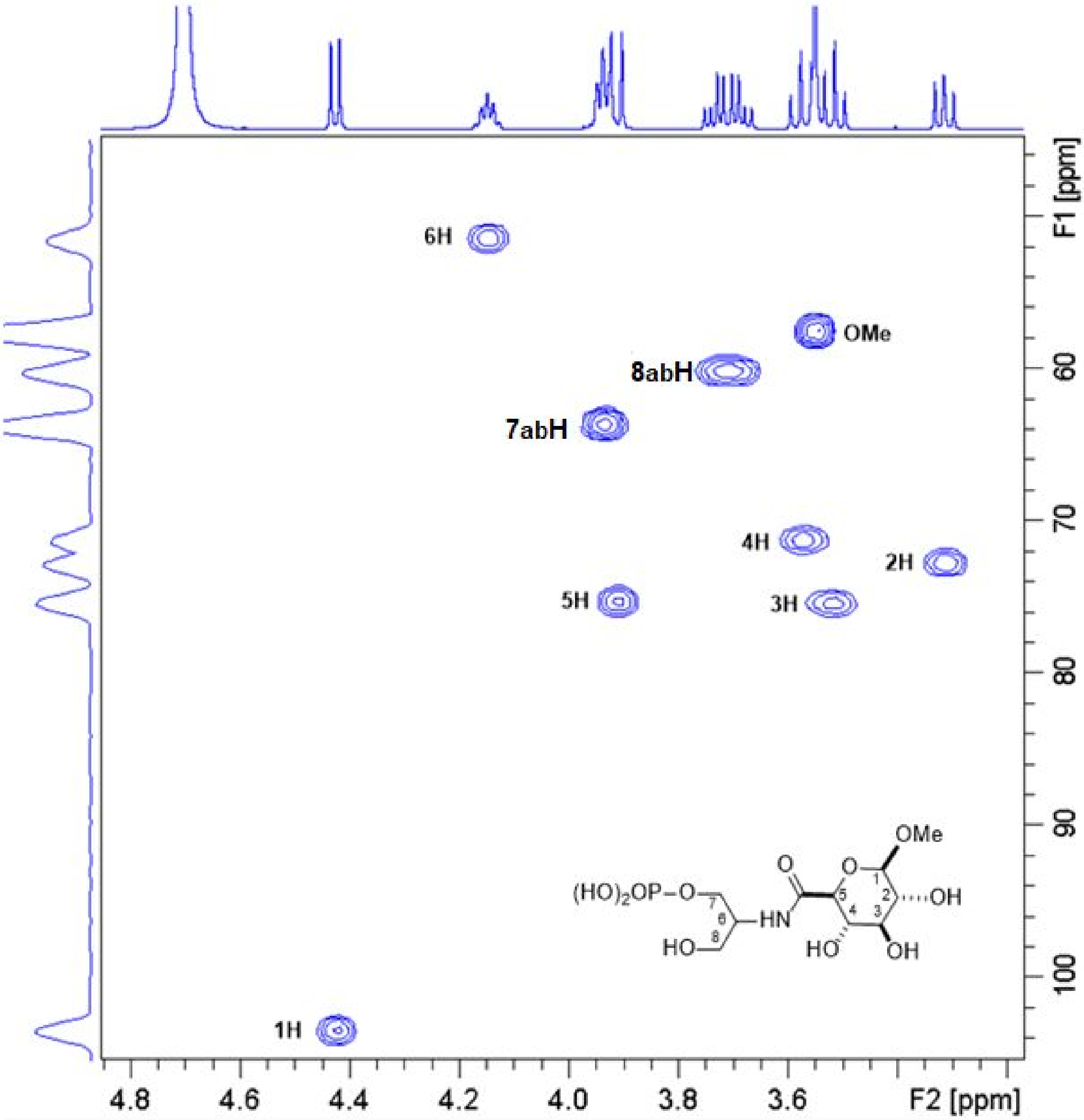

Figure S7. HSQC 2D spectrum of compound **2a** in D_2O. In compound **2a**, the ^{13}C NMR signals corresponding to C-1 to C-5 were observed at δ 103.5, 72.8, 75.4, 71.4 and 75.4 ppm, respectively.

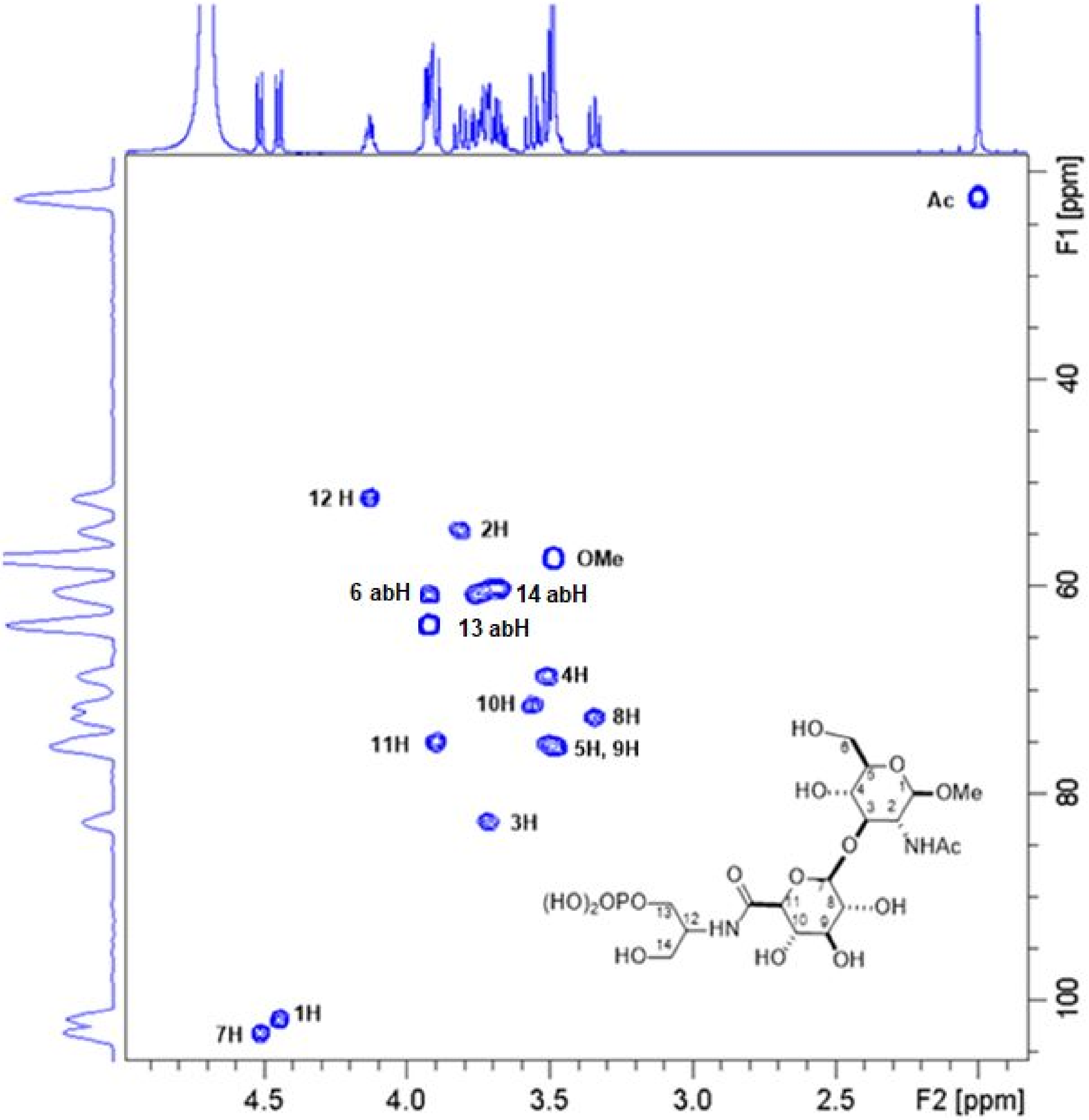

Figure S8. HSQC 2D spectrum of compound **7** in D_2O. In compound **7**, ^{13}C NMR signals corresponding to C-3, C-4, and C-6 resonate at δ 82.6, 68.5, and 60.7 ppm

Assignment of the glycosidic linkage in compound 7

The position of the glycosidic linkage in **compound 7** was determined to distinguish among possible (1→3), (1→4), or (1→6) connections. For this purpose, the ^{1}H and ^{13}C NMR assignments of compounds **2a** and **5** were established using ^{1}H NMR, COSY, and HSQC experiments. In compound **5**, the ^{13}C NMR signals corresponding to C-3, C-4, and C-6 were observed at δ 74.0, 70.1, and 60.9 ppm, respectively. In the disaccharide product **7**, these carbons resonate at δ 82.6, 68.5, and 60.7 ppm, respectively. A pronounced downfield shift is observed for C-3 (δ 74.0 → δ 82.6), consistent with its involvement in glycosidic bond formation. In contrast, C-4 exhibits a small upfield shift (δ 70.1 → δ 68.5), while C-6 remains largely unchanged (δ 60.9 → δ 60.7).

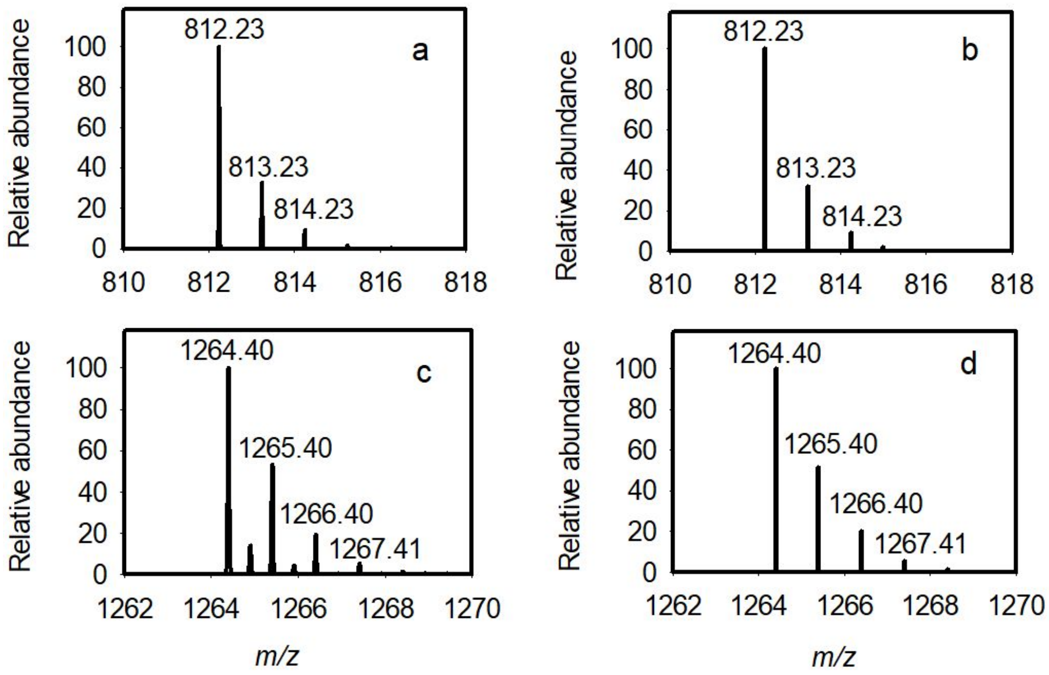

Figure S9. ESI mass spectral data for the [M-H$^+$]$^-$ anion of the isolated oligomers **9** and **11**. (**a**) experimental data for oligomer **9**; (**b**) predicted distribution for oligomer **9**; (**c**) experimental data for oligomer **11**; (**d**) predicted distribution for oligomer **11**.

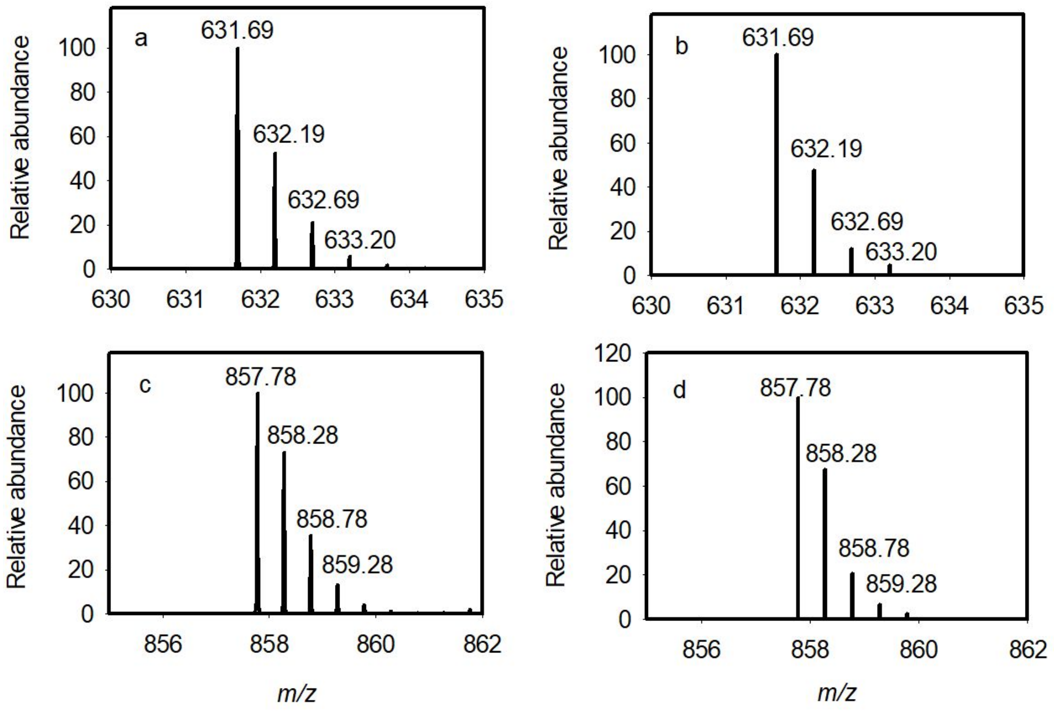

Figure S10. ESI mass spectral data for the $[M\text{-}2H^+]^{2-}$ anion of the isolated oligomers **11** and **13**. (**a**) experimental data for oligomer **11**; (**b**) predicted distribution for oligomer **11**; (**c**) experimental data for oligomer **13**; (**d**) predicted distribution for oligomer **13**.

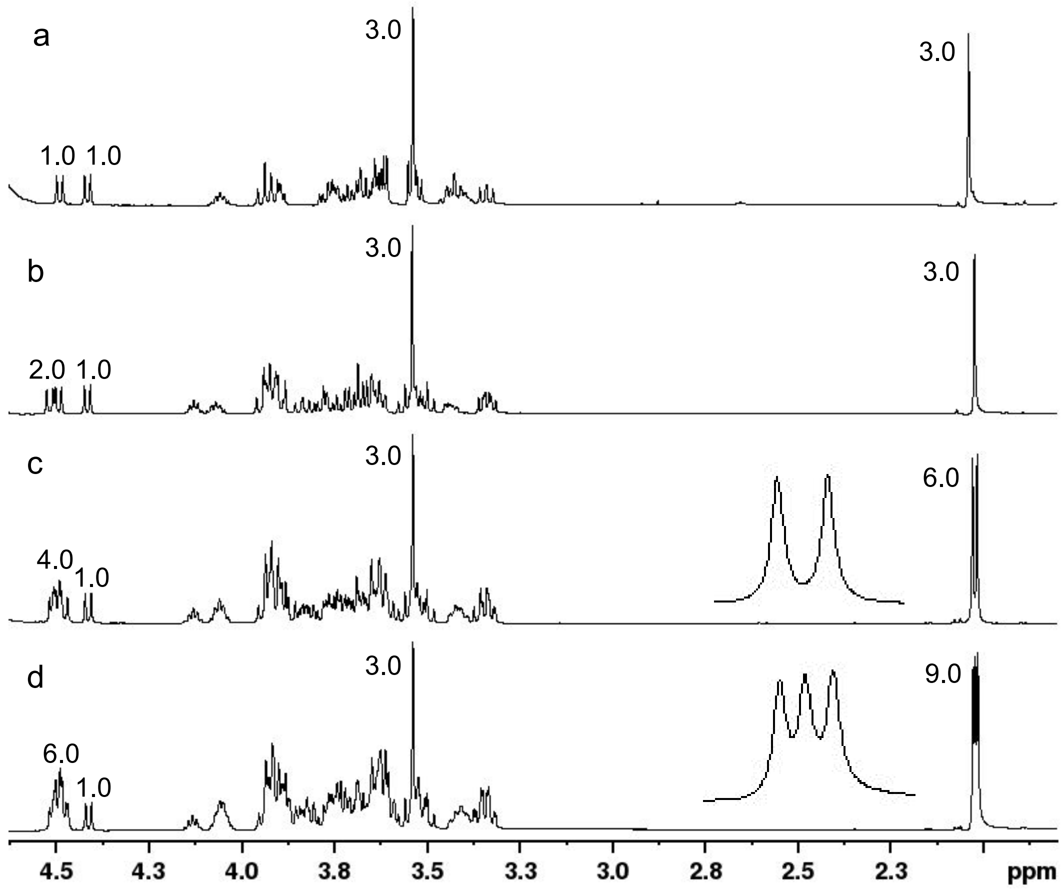

Figure S11. ^{1}H NMR spectra of isolated oligomers. (**a**) dimer **4a**; (**b**) trimer **9**; (**c**) pentamer **11**; (**d**) heptamer **13**. The inset highlights the separate resonances at ~2.1 ppm for the methyl groups in the NAc-substituents in pentamer **11** and heptamer **13**.

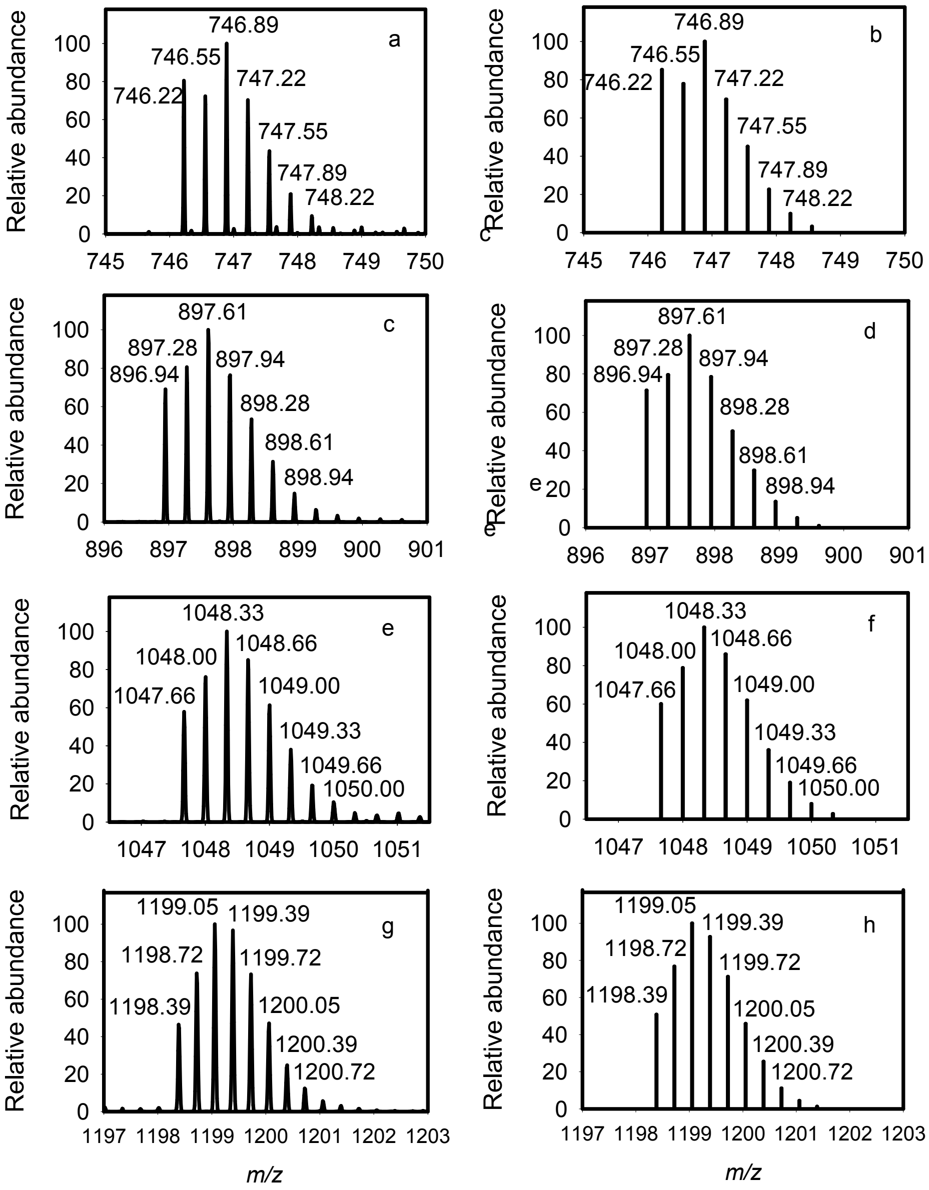

Figure S12. Expanded views of the isotopic clusters for the nanomer **15-2**, undecamer **15-3**, tridecamer **15-4**, and pentadecamer **15-5** oligomeric products identified in **Figure 9** (z = 3). (**a**) experimental data for oligomer **15-2**; (**b**) predicted distribution for oligomer **15-2**; (**c**) experimental data for oligomer **15-3**; (**d**) predicted distribution for oligomer **15-3**; (**e**) experimental data for oligomer **15-4**; (**f**) predicted distribution for oligomer **15-4**; (**g**) experimental data for oligomer **15-5**; (**h**) predicted distribution for oligomer **15-5**.

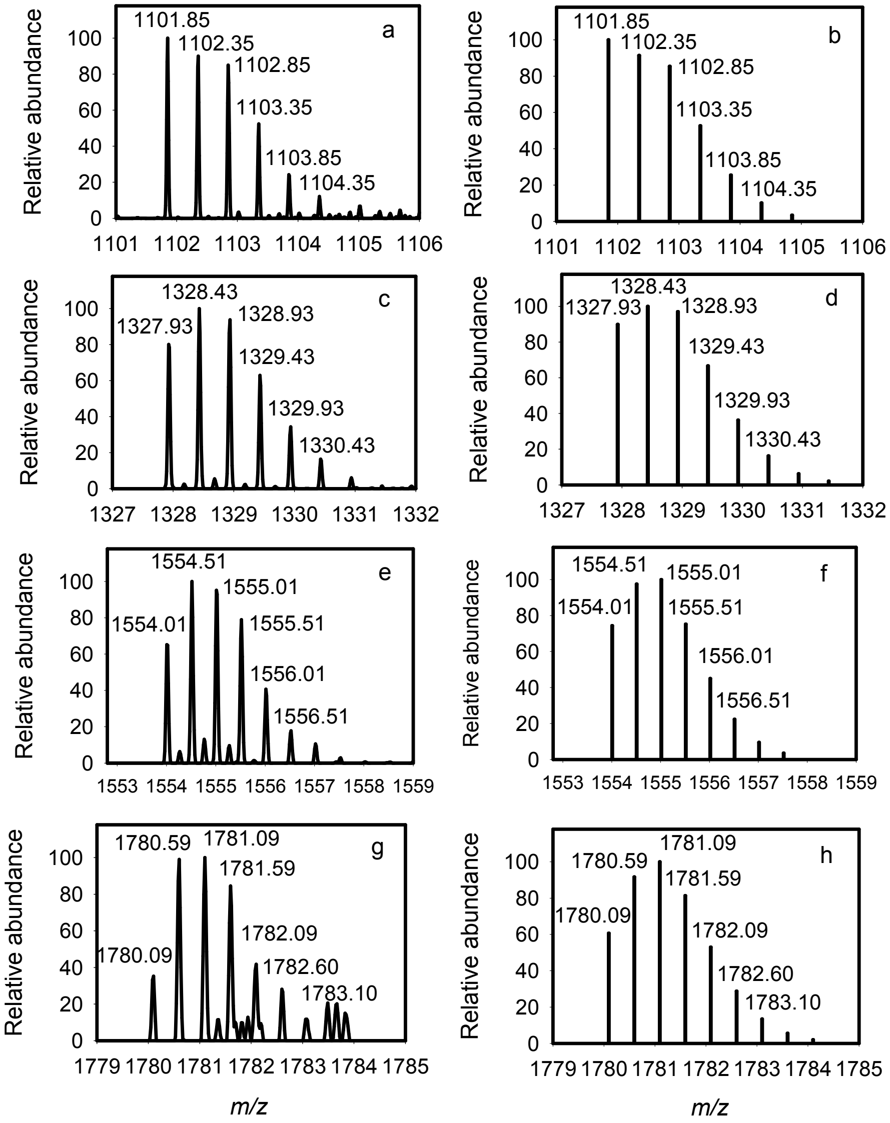

Figure S13. Expanded views of the isotopic clusters for the nanomer **15-2**, undecamer **15-3**, tridecamer **15-4**, and pentadecamer **15-5** oligomeric products identified in **Figure 9** (z = 2). (**a**) experimental data for oligomer **15-2**; (**b**) predicted distribution for oligomer **15-2**; (**c**) experimental data for oligomer **15-3**; (**d**) predicted distribution for oligomer **15-3**; (**e**) experimental data for oligomer **15-4**; (**f**) predicted distribution for oligomer **15-4**; (**g**) experimental data for oligomer **15-5**; (**h**) predicted distribution for oligomer **15-5**;

Table S1. Mass spectral data for enzyme synthesized trimer **9**, pentamer **11**, and heptamer **13**.

**Oligomer**	**Formula**	**Theoretical Mass** $[M-H^+]^-$ *m/z* $z = 1$	**Relative Abundance**	**Measured Mass** $[M-H^+]^-$ *m/z* $z = 1$	**Relative Abundance**
trimer **9**	$C_{27}H_{48}N_3O_{23}P$ mol. wt. 813.65 amu	812.23	100.00	812.23	100.00
		813.23	31.72	813.23	32.90
		814.23	9.60	814.23	9.69
pentamer **11**	$C_{44}H_{76}N_5O_{35}P$ mol. wt. 1266.07 amu	1264.40	100.00	1264.40	100.00
		1265.40	51.61	1265.40	53.34
		1266.40	20.25	1266.40	19.40
		1267.41	5.87	1267.41	5.62
heptamer **13**	$C_{61}H_{104}N_7O_{47}P$ mol. wt. 1718.48 amu	1716.56	100.00	1716.56	100.00
		1717.56	71.51	1717.56	84.20
		1718.56	34.86	1718.56	35.70
		1719.56	12.74	1719.56	11.11
**Oligomer**	**Formula**	**Theoretical Mass** $[M-2H^+]^{2-}$ *m/z* $z = 2$	**Relative Abundance**	**Measured Mass** $[M-2H^+]^{2-}$ *m/z* $z = 2$	**Relative Abundance**
pentamer **11**	$C_{44}H_{76}N_5O_{35}P$ mol. wt. 1266.07 amu	631.69	100.00	631.69	100.00
		632.19	52.33	632.19	47.62
		632.69	20.90	632.69	11.90
		633.20	5.97	633.20	4.76
heptamer **13**	$C_{61}H_{104}N_7O_{47}P$ mol. wt. 1718.48 amu	857.78	100.00	857.78	100.00
		858.28	72.86	858.28	67.46
		858.78	35.64	858.78	20.63
		859.28	13.01	859.28	6.35

Table S2. Mass spectral data for enzyme synthesized oligomers.

Oligomer	Formula	Theoretical Mass $[M\text{-}H^{+}+2Cl^{-}]^{3-}$ *m/z* *z* = 3	Relative Abundance	Measured Mass $[M\text{-}H^{+}+2Cl^{-}]^{3-}$ *m/z* *z* = 3	Relative Abundance
nonamer (**15-2**)	$C_{78}H_{132}N_9O_{59}P$ mol. wt. 2170.89 amu	746.22	85.18	746.22	80.48
		746.55	77.84	746.55	72.31
		746.89	100.00	746.89	100.00
		747.22	69.73	747.22	70.32
		747.56	45.03	747.56	43.34
		747.89	22.65	747.89	20.94
		748.22	9.87	748.22	9.34
undecamer (**15-3**)	$C_{95}H_{160}N_{11}O_{71}P$ mol. wt. 2623.30 amu	896.94	71.45	896.94	69.08
		897.28	79.52	897.28	80.54
		897.61	100.00	897.61	100.00
		897.94	78.46	897.94	76.36
		898.28	50.17	898.28	53.42
		898.61	29.87	898.61	31.31
		898.94	13.52	898.94	14.82
tridecamer (**15-4**)	$C_{112}H_{188}N_{13}O_{83}P$ mol. wt. 3075.72 amu	1047.66	60.08	1047.66	57.85
		1048.00	78.82	1048.00	76.10
		1048.33	100.00	1048.33	100.00
		1048.66	85.97	1048.66	85.00
		1049.00	61.93	1049.00	61.30
		1049.33	36.02	1049.33	37.97
		1049.66	19.00	1049.66	19.13
		1050.00	7.98	1050.00	10.40
pentadecamer (**13-5**)	$C_{129}H_{216}N_{15}O_{95}P$ mol. wt. 3528.13 amu	1198.39	50.79	1198.39	46.37
		1198.72	76.74	1198.72	73.71
		1199.05	100.00	1199.05	100.00
		1199.39	92.70	1199.39	96.71
		1199.72	71.18	1199.72	73.24
		1200.05	45.89	1200.05	47.12
		1200.39	25.46	1200.39	12.35
		1200.72	5.32	1200.72	5.62

Table S3. Mass spectral data for enzyme synthesized oligomers.

Oligomer	Formula	Theoretical Mass $[M-H^{+}+Cl^{-}]^{2-}$ m/z z = 2	Relative Abundance	Measured Mass $[M-H^{+}+Cl^{-}]^{2-}$ m/z z = 2	Relative Abundance
nonamer (**15-2**)	$C_{78}H_{132}N_9O_{59}P$ mol. wt. 2170.89 amu	1101.85	100.00	1101.85	100.00
		1102.35	91.40	1102.35	90.05
		1102.85	85.43	1102.85	85.05
		1103.35	52.63	1103.35	52.38
		1103.85	25.54	1103.85	24.15
		1104.35	10.11	1104.35	12.08
undecamer (**15-3**)	$C_{95}H_{160}N_{11}O_{71}P$ mol. wt. 2623.30 amu	1327.93	89.85	1327.93	80.05
		1328.43	100.00	1328.43	100
		1328.93	97.00	1328.93	93.81
		1329.43	66.67	1329.43	62.95
		1329.93	36.26	1329.93	34.27
		1330.43	16.24	1330.43	16.36
tridecamer (**15-4**)	$C_{112}H_{188}N_{13}O_{83}P$ mol. wt. 3075.72 amu	1554.01	74.38	1554.01	65.13
		1554.51	97.58	1554.51	100
		1555.01	100	1555.01	95.13
		1555.51	75.21	1555.51	78.94
		1556.01	45.04	1556.01	40.66
		1556.51	22.36	1556.51	17.72
pentadecamer (**15-5**)	$C_{129}H_{216}N_{15}O_{95}P$ mol. wt. 3528.13 amu	1780.09	60.64	1780.09	35.24
		1780.59	91.63	1780.59	99.05
		1781.09	100	1781.09	100
		1781.59	81.37	1781.59	84.53
		1782.09	52.99	1782.09	41.71
		1782.60	28.76	1782.60	28.05
		1783.10	13.44	1783.10	11.74
